# ﻿Study of terrestrial diatoms in corticolous assemblages from deciduous trees in Central Europe with descriptions of two new *Luticola* D.G.Mann taxa

**DOI:** 10.3897/phytokeys.221.95248

**Published:** 2023-03-07

**Authors:** Mateusz Rybak, Paweł Czarnota, Teresa Noga

**Affiliations:** 1 Department of Agroecology and Forest Utilization, University of Rzeszów, ul. Ćwiklińskiej 1a, 35-601, Rzeszów, Poland University of Rzeszów Rzeszów Poland; 2 Department of Ecology and Environmental Protection, University of Rzeszów, ul. Zelwerowicza 4, 35–601, Rzeszów, Poland University of Rzeszów Rzeszów Poland; 3 Department of Soil Studies, Environmental Chemistry and Hydrology, University of Rzeszów, ul. Zelwerowicza 8B, 35-601, Rzeszów, Poland University of Rzeszów Rzeszów Poland

**Keywords:** Bacillariophyceae, bark chemistry, bryosphere, corticolous habitats, diversity, taxonomy

## Abstract

Although many studies have examined the algae associated with various habitats in tree trunks, the diatoms in these environments are still poorly studied. Studies of corticolous algae mainly focus on green algae and cyanobacteria, which are usually immediately visible, while diatoms are mostly overlooked or not reported. During the research, 143 species of diatoms were identified, including two new representatives of the genus *Luticola: L.bryophila***sp. nov.** with relatively large central area and short distal raphe endings and *L.confusa***sp. nov.** characterized by the presence of small depressions on central raphe endings. Both are described herein based on light and scanning electron microscopy observations and compared to similar taxa based on literature data. Basic morphological data for almost all the diatom taxa are noted, and their habitat requirements, and photographic documentation are also presented. The present research showed that the occurrence of diatom assemblages on tree trunks is influenced by various factors like host tree species, the area where the host tree grows, and the availability of suitable microhabitats within the trunk. However, the species composition of this assemblages depends mainly on the tree species.

## ﻿Introduction

Despite over a century of study on terrestrial algae (West and West 1894; Fritsch 1907; Petersen 1915, 1928, 1935; Beyens 1989), less is still known about these ecological groups than is known about algae from aquatic environments. Most research on terrestrial algae is floristic (Dodd and Stoermer 1962; Schlichting 1975; Camburn 1983; McMillan and Rushforth 1985; Hoffmann 1989), while few works focus on their ecology (Ress 2012; Foets et al. 2021).

Many different terms are used to name algal assemblage in their terrestrial environments depending on environmental conditions and available water sources (Ress 2012; Furey et al. 2020). Bark-associated assemblages that inhabit raised objects that receive moisture exclusively from the atmosphere are categorized as euaerial. The terms epiphylophyte and epiphloeophyte are used to refer to bark-associated algae. The former describes algae growing on leaf surfaces, while the latter describes algae inhabiting tree bark often in association with bryophytes and lichens (Chapman and Chapman 1973; Awasthi 2015; Sivakumar 2016). The term corticophiles is also sometimes used in reference to algal communities growing directly on tree barks (Kawecka and Eloranta 1994; Bhakta et al. 2014; Narasimha Rao 2017).

Bark surfaces create special microclimatic niches for algae. Thanks to cracks, they retain moisture, protect against wind, and provide shade and nutrients that are compounds from accumulated dust which dissolve in rainwater (Kawecka and Eloranta 1994). Algae inhabiting tree trunks have been the subject of a few studies, most of which focused on green algae and cyanobacteria (Foerester 1971; Wylie and Schlichting 1973; Mrozińska 1990; Thompson and Wujek 1997; Salleh and Milow 1999; Czerwik and Mrozińska 2000; Neustupa 2003, 2005; Soni and Shukla 2006; Neustupa and Škaloud 2008, 2010; Lemes-Da-Silva et al. 2010; Bhakta et al. 2014; Kharkongor and Ramanujam 2014; Štifterová and Neustupa 2015; Narasimha Rao 2017; Ambika and Krishnamurthy 2018). In research on bark-associated algae, diatoms have been reported rarely to date and are usually only listed as single taxa in species’ lists (Neustupa and Škaloud 2010; Neustupa and Štifterová 2013; Kharkongor and Ramanujam 2014; Štifterová and Neustupa 2015; Nirmala et al. 2016; Eldrin 2019). Lakatos and co-authors (Lakatos et al. 2004) studied diatoms inhabiting the thallus of the arboreal lichen *Coenogoniumlinkii* Ehrenb., and during this research they noted 18 taxa. Research conducted by Qin et al. (2016) was focused on diatoms associated with arboreal bryophytes growing on trees in Wuhan, and they collected samples mainly from *Cinnamomumcamphora* Ness & Eberm and they noted 76 taxa represented 13 genera. Studies on diatoms inhabiting arboreal mosses from the Indo-Burma biodiversity hot spot showed the presence of 56 species from 21 genera (Cheran et al. 2022). The only work from Europe is based on preliminary results from samples collected from a single tree Despite the small number of samples, 47 species have been identified (Rybak et al. 2018). In all these works, each studied sample contained only few species (usually fewer than 20) and were dominated by species from genera like: *Luticola*, *Humidophila*, *Pinnularia* and *Orthoseira*.

The aim of the study was to investigate the taxonomic diversity and ecological requirements of diatoms inhabiting various microhabitats on trunks of deciduous trees in Central Europe in areas confronting various degrees of human impact. Additionally, preferences of diatoms for host tree species and microhabitats within trunks were determined.

## ﻿Methods

**Figure 1.**
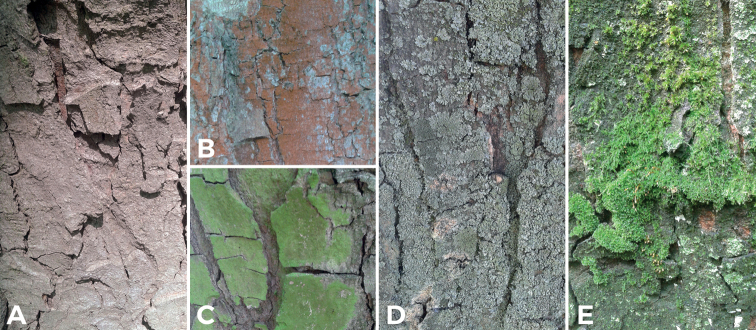
Types of studied microhabitats on the example of sycamore maple. Bare bark (**A**), bark covered by green algae (**B, C**), bark with lichen covering (**D**) and bark covered by bryophytes (**E**).

Samples were collected in 2017 and 2018 four times each year in the first half of April and June, and the second half of August and October from heights of 20 cm (referred to as trunk base) and 150 cm above ground level from several tree trunk microhabitats, i.e., bare bark, moss clumps, bark covered with lichens, bark with visible mats of algae (Fig. 1), at eight sampling sites and from the same trees of the following taxa: sycamore maple (*Acerpseudoplatanus* L.), linden (*Tilia* spp. L.), and poplar (*Populus* spp. L.). The designated sites included city centers (sites 1 and 4), small peripheral estates (sites 2 and 5), park complexes in suburban areas (sites 3 and 6), and the buffer zones of two national parks: Magurski National Park (site 7) and Gorczański National Park (site 8) (Table 1). Samples in form of bark pieces, together with overgrowing epiphytes, were chipped off using a hammer and chisel and placed in paper envelopes to avoid mold. During the collection of materials, no visible signs of the presence of diatoms (like slime or different color stains) were noted on the surface.

**Table 1.** List of studied sites with the sampled tree taxa and microhabitat type over their trunk.

Site	Tree species	Coordinates	Studied microhabitat
1	*Acerpseudoplatanus* L.	50°34'09.1"N, 22°03'57.5"E	bare bark, lichens
	*Tiliaplatyphyllos* Scop.	50°34'04.1"N, 22°04'01.9"E	bare bark, lichens
	*Populusnigra* L. ‘Italica’	50°34'08.5"N, 22°03'56.8"E	bare bark, mosses, lichens
2	*Acerpseudoplatanus* L.	50°34'12.1"N, 22°04'20.4"E	lichens
	*Tiliacordata* Mill.	50°34'12.2"N, 22°04'20.8"E	lichens
	*Populusnigra* L.	50°34'16.4"N, 22°04'18.6"E	bare bark, mosses, lichens
3	*Acerpseudoplatanus* L.	50°36'13.2"N, 22°02'01.7"E	bare bark, mosses, green algae mats
	*Tiliacordata* Mill.	50°35'58.2"N, 22°01'49.7"E	bare bark, mosses
	*Populusnigra* L.	50°35'59.0"N, 22°01'49.6"E	bare bark, mosses
4	*Acerpseudoplatanus* L.	50°01'06.7"N, 22°01'01.5"E	bare bark, lichens
	*Tiliaplatyphyllos* Scop.	50°01'18.4"N, 22°00'56.7"E	bare bark, lichens
	*Populusnigra* L. ‘Italica’	50°01'18.8"N, 22°01'00.6"E	bare bark, mosses, lichens
5	*Acerpseudoplatanus* L.	50°00'08.9"N, 22°01'54.1"E	bare bark, mosses
	*Tiliacordata* Mill.	50°00'11.0"N, 22°01'54.1"E	bare bark, mosses
	*Populusnigra* L.	50°00'09.5"N, 22°01'48.3"E	bare bark, mosses, lichens, green algae mats
6	*Acerpseudoplatanus* L.	50°00'36.3"N, 21°59'16.0"E	bare bark, mosses
	*Tiliacordata* Mill.	50°00'39.1"N, 21°59'20.5"E	bare bark, mosses
	*Populusalba* L.	50°00'42.6"N, 21°59'23.6"E	bare bark, mosses, green algae mats
7	*Acerpseudoplatanus* L.	49°30'10.3"N, 21°29'31.1"E	bare bark, mosses, green algae mats
	*Tiliacordata* Mill.	49°30'42.8"N, 21°29'57.0"E	bare bark, mosses, lichens
	*Populustremula* L.	49°30'58.1"N, 21°29'38.3"E	bare bark, mosses
8	*Acerpseudoplatanus* L.	49°37'01.8"N, 20°04'07.0"E	bare bark, mosses, lichens
	*Tiliacordata* Mill.	49°37'01.4"N, 20°04'06.7"E	bare bark, mosses, lichens
	*Populusnigra* L.	49°37'02.8"N, 20°04'14.3"E	bare bark, mosses, lichens

The samples were also used to prepare filtrates for pH and conductivity analyses. The filtrates were obtained by soaking bare bark pieces in deionized water (1:10 by weight) for 24 hours. The intact pieces of bark were used to obtain a solution similar to that forming on the bark surface that is a source of water and nutrients for corticolous organisms; in the case of trees completely covered by epiphytic mosses, the material together with them was used to obtain filtrates. Electrolytic conductivity and pH were measured with a MARTINI pH56 pH meter and a MARTINI EC59 conductometer (Milwaukee Electronics Kft.). The ions’ content was determined using a Thermo scientific DIONEX ICS–5000+DC device in the Departmental Laboratory of Analysis of Environmental Health and Materials of Agricultural Origin at the University of Rzeszów.

A modified method by Qin et al. (2016) was used to obtain clean diatom material. For the purpose of separating bryophytes and diatoms from the bark surface, a part of the material collected was placed in beakers to which 50 ml 30% hydrogen peroxide (H_2_O_2_) was added, and these were left at room temperature for 48 hours. In the next step, bark fragments were rinsed with deionized water, and the resulting solution was collected in the same beaker in which the bark was digested. To obtain clean diatom valves, the solution was centrifuged to remove excess hydrogen peroxide and again digested in a mixture of sulfuric acid (H_2_SO_4_) and potassium dichromate (K_2_Cr_2_O_7_) until the organic matter was completely dissolved. In the last step, the burning mixture was removed by centrifugation with distilled water (at 2500 rpm).

Light microscope slides were prepared by applying the cleaned diatom suspension to cover-slips that were left to dry. The dried material was mounted in synthetic Pleurax resin, Brunel Microscopes ltd. (refractive index 1.75). To better define the species composition of the assemblages analyzed, two microscope samples on coverslips were mounted on a single slide. In total, 647 samples were collected and analyzed. The diatoms were identified under a Carl Zeiss Axio Imager.A2 light microscope (LM) with a Zeiss AxioCam ICc 5 camera at 1000× magnification. For Scanning Electron Microscope (SEM) observations, the samples were coated in a Turbo-Pumped Sputter Coater Quorum Q 150OT ES with a 20 nm layer of gold and viewed under a Hitachi SU 8010 microscope.

Two microscope slides were made from each collected sample. Diatoms were identified in both slides by observations in all possible adjacent transects. During species identification, all identified valves were counted until a number of 400 was reached. Identification of species was continued for species composition also after reaching the assumed limit of 400 valves. The dominance structure and similarity analysis were determined only for samples for which a minimum of 200 valves were counted. Species with a minimum share of 10% in the assemblages were considered dominants. The remaining samples were considered unrepresentative because of the insufficient development of assemblages or their complete absence. To present the morphological variability of the observed taxa, valves dimensions were measured under light microscope using AxioVision SE62 Rel. 4.9.1 software. For range dimension of commonly occurred taxa ca. 50 specimens were measured including the biggest and the smallest observed specimens. In the case of rare taxa (observed in <10 samples) each observed specimen was measured.

Diatom diversity was analyzed using the Shannon diversity index (H’) and the Evenness index (J’). Principal Component Analysis (PCA) was performed to determine the similarity of diatom assemblages. Prior to analysis, diatom data were square root transformed. Redundancy analysis (RDA, gradient length = 1.9 SD) was performed to determine the effect of bark chemistry on diatom assemblages, but none of the parameters showed statistically significant effects on the assemblages studied (p> 0.05). All analyses (PCA, RDA) were performed using Canoco 5 (Ter Braak and Šmilauer 2012).

Student’s t-test was used to analyze the significance of differences in the chemical parameters of the samples, and values at p<0.05 were considered statistically significant. All calculations were performed using Statistica 13.3 software.

Diatom terminology and identification were based on Round et al. (1990), Krammer and Lange-Bertalot (1986, 1988, 1991a, b), Krammer (2000), Lange-Bertalot (1993), Lange-Bertalot et al. (2003, 2011, 2017), Levkov et al. (2013, 2017, 2019) and Houk et al. (2017).

## ﻿Results

### ﻿Chemical analyses

The pH of the analyzed filtrates indicated slightly acid to neutral condition of the barks of the tree species examined. The electrolytic conductivity values measured in the filtrates of all the trees analyzed indicated a very wide range (from 49 µm cm^-1^ to 5 846 µS cm^-1^), regardless of sampling height (Table 2). Statistically significant differences in the parameters measured for samples taken from heights of 20 and 150 cm were noted for pH in linden and poplar and for electrolytic conductivity in sycamore maple. On the other hand, the analysis of chemical parameters of permeates depending on tree species was not statistically significantly different. The parameters measured did not differ significantly among the tree species studied (Table 2).

**Table 2.** Chemical parameters measured in filtrates obtained from bark of studied trees, given range (minimum and maximum), and median (brackets), bold indicates value for samples from trunk bases. * – indicates a parameter in which differences between the studied heights were statistically significant (p> 0.05).

Tree taxon	*Acer*	*Tilia*	*Populus*
**pH**	5.3–7.3 (6.3)	4.7–7.3 (6.0)*	4.8–7.5 (6.3)*
	**4.8–7.5 (6.4)**	4.8–7.5 (6.4)*	5.1–7.4 (6.5)*
**Cond. [μS cm^–1^**]	49–2305 (167)*	49–462 (202)	52–2305 (323.1)
	64–604 (228)*	**69–5846 (338)**	**39–1199 (332.1)**
**Cl- [mg/l**]	0.5–29.9 (3.3)	1.1–20.7 (6.3)	0.442–103.7 (9.4)
	**0.5–14.5 (3.3)**	**0.2–71.7 (9.0)**	**0.232–98.6 (8.8)**
**NO_3_- [mg/l**]	<0.001–4.6 (1.5)	<0.001–4.6 (2.5)	<0.001–159.3 (3.4)
	**<0.001–16.5 (1.2)**	**<0.001–89.9 (5.2)**	**<0.001–42.2 (2.3)**
**PO_4_^3-^ [mg/l**]	<0.001–44.8 (11.1)	<0.001–34.8 (10.1)*	<0.001–36.7 (8.8)*
	**2.7–42.5 (14.1)**	<0.001–39.2 (16.3)*	<0.001–64.0 (14.3)*
**SO_4_^2-^ [mg/l**]	0.7–55.5 (7.2)	<0.001–61.5 (18.6)	<0.001–400.9 (20.9)
	**0.9–14.9 (6.5)**	**0.6–3 278.2 (66.7)**	**0.2–10.7 (20.8)**
**Na^+^ [mg/l**]	0.3–12.4 (2.5)	0.4–12.2 (3.1)*	0.273–59.2 (6.8)
	**0.3–18.5 (3.4)**	0.3–21.0 (5.7)*	**0.3–55.3 (7.4)**
**K^+^ [mg/l**]	10.2–137.9 (33.7)	9.509–68.1 (35.9)	7.8–752.2 (86.3)
	**11.2–161.4 (38.4)**	**<0.001–1510.2 (65.8)**	**<0.001–386.3 (75.2)**
**Ca^2+^ [mg/l**]	0.6–48.2 (6.7)	0.1–26.4 (10.6)	0.3–56.5 (8.5)
	**0.2–34.7 (8.0)**	**0.3–317.7 (16.4)**	**0.3–28.9 (8.7)**
**Mg^2+^ [mg/l**]	0.2–18.5 (2.4)	0.3–10.6 (2.7)	0.1–37.1 (3.5)
	**0.1–14.0 (2.6)**	**0.6–62.8 (3.8)**	**0.2–10.7 (3.0)**
**NH_4_^+^ [mg/l**]	<0.001–28.5 (3.8)*	0.1–23.7 (5.6)	<0.001–12.3 (2.3)*
	0.7–32.8 (7.3)*	**<0.001–228.9 (12.7)**	<0.001–103.0 (5.8)*

## ﻿Taxonomic results

### ﻿Description of new species

#### ﻿ Luticola bryophila

Taxon classificationPlantaeNaviculalesDiadesmidaceae

M.Rybak, Czarnota & Noga sp. nov.

D34B9000-5EFB-552C-9588-B15D18735ADD

Fig. 2

##### Description.

Rectangular in girdle view. Valves linear to linear lanceolate with weakly protracted and moderately rounded apices, smaller specimens without protracted apices. Valve length 10–25 µm, and valve width 4–6 µm. Axial area narrow linear, slightly expanded near central area. Central area wide and rounded, bordered by 3–4 areolae. Ghost areolae commonly present in central area. Round isolated pore located half way between valve center and margin. Raphe branch straight with both endings clearly curved to site opposite to isolated pore. Distal raphe endings short, not continuing onto mantle. Internal raphe slit simple and straight, distal endings form slightly developed helictoglossae. Striae number 18–20 in 10 µm composed of 2–3 same sized, rounded to slightly elongated areolae. Single row of areolae present also on valve mantle. Internally areolae covered by hymen forming continuous strip, separated by not thickened virgae. Internally small lipped opening of isolated pore visible. Marginal channel located on valve face/mantle junction occluded by hymenes and visible internally.

**Figure 2.**
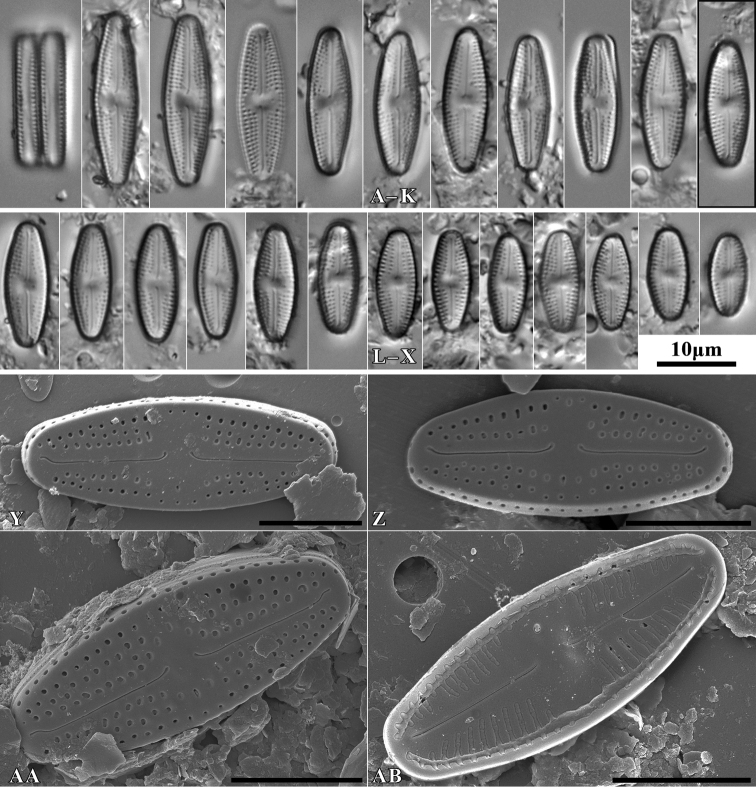
*Luticolabryophila* sp. nov., LM microphotographs of girdle view (**A**) and size diminution series (**B–X**), black frame indicate the holotype specimen. SEM microphotographs (**Y–AB**) of valve in external (**Y–AA**) and internal view (**AB**). Scale bars: 10 µm (**A–X**); 5 µm (**Y–AB**).

##### Type material.

**Figure 3.**
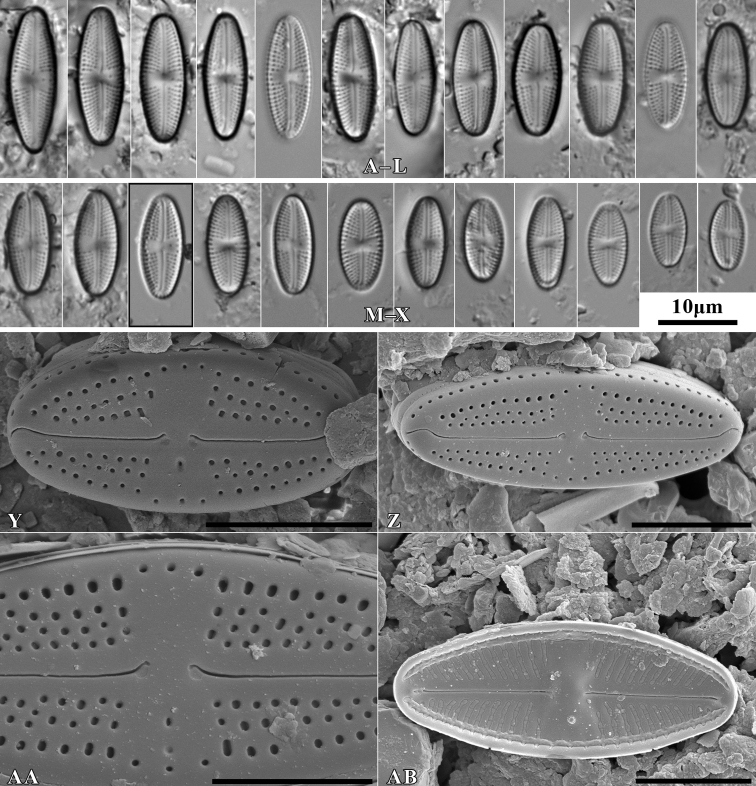
*Luticolaconfusa* sp. nov., LM microphotographs of size diminution series (**A–X**), black frame indicate the holotype specimen. SEM microphotographs (**Y–AB**), valve in external (**Y–AA**) and internal view (**AB**). Scale bars: 10 µm (**A–X**); 5 µm (**Y, Z, AB**),

***Holotype***: Slide SZCZ 28844 and unmounted material with the same number stored in A. Witkowski Diatom Collection of the Institute of Marine and Environmental Sciences, University of Szczecin, holotype specimen designated here in Fig. 3K

***Isotype***: Slide no. 2017/18 and unmounted material with the same number at the University of Rzeszów, Poland.

##### Type locality.

Stalowa Wola, Podkarpacie Province, Poland, 50°34'16.4"N, 22°04'18.6"E, leg. M. Rybak.

##### Etymology.

The name refers to the occurrence of the species mainly in terrestrial bryophytes.

##### Distribution.

Species observed at most of the sites studied, always in single specimens, mainly in samples of bryophytes from trunk bases.

##### Similar species.

*Luticolasparsipunctata* Levkov, Metzeltin & Pavlov (Levkov et al. 2013, p. 222); *Luticolatenuis* Levkov, Metzeltin & Pavlov (Levkov et al. 2013, p. 236).

#### ﻿ Luticola confusa

Taxon classificationPlantaeNaviculalesDiadesmidaceae

M.Rybak & Czarnota sp. nov.

1BBE7024-59B3-5923-9218-7B1FA76954E3

Fig. 3

##### Description.

Valves elliptic to elliptic-lanceolate with rounded apices, rectangular in girdle view. Valve length 9–22 µm, and valve width 4.5–5.5 µm. Axial area narrow and linear, central area elliptic bordered by 3–4 areolae. Round isolated pore located halfway between valve center and margin. Raphe branch straight. Proximal raphe endings deflected to site opposite to stigma with small rounded depressions. Distal raphe endings hooked continuing onto valve mantle. Internally raphe slit simple and straight, distal endings form slightly developed helictoglossae. Striae number 20–22 in 10 µm composed mainly of 4 areolae, single row of areolae also present on valve mantle. On apices row of mantle areolae interrupted by distal raphe endings. Internally areolae covered by hymen forming continuous strip, separated by not thickened virgae. Internally small lipped opening of isolated pore visible. Marginal channel located on valve face/mantle junction occluded by hymenes and visible internally.

##### Type material.

**Figure 4.**
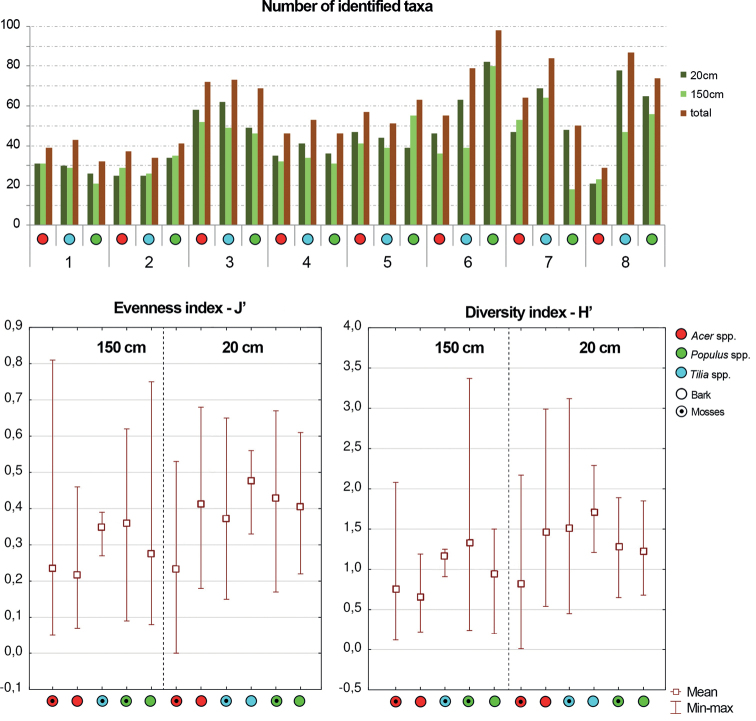
Number of diatom taxa identified during the research on each of the examined trees on each site and value of Eveness index (J’) and Shannon index (H’). 1–8 number of sampling site.

***Holotype***: Slide SZCZ28845 and unmounted material with the same number stored in A. Witkowski Diatom Collection of the Institute of Marine and Environmental Sciences, University of Szczecin, holotype specimen designated here in Fig. 4O.

***Isotype***: Slide no. 2018/454 and unmounted material with the same number at the University of Rzeszów, Poland.

##### Type locality.

Stalowa Wola, Podkarpacie Province, Poland, 50°36'13.2"N, 22°02'01.7"E, leg. M. Rybak.

##### Etymology.

The name refers to possible past confusions in identification of the species described with other small taxa with elliptic-lanceolate valves.

##### Distribution.

Species observed at all sites studied, always in the form of individual specimens. It mainly occurred in samples taken from the base of the trunks of the trees studied.

##### Similar species.

*Luticolaimbricata* (W.Bock) Levkov, Metzeltin and Pavlov (Levkov et al. 2013, p. 134); *L.pseudoimbricata* Levkov, Metzeltin & Pavlov (Levkov et al. 2013, p. 134); *L.obscura* Levkov, Tofilovska, C.E.Wetzel, Mitić-Kopanja & Ector (Levkov et al. 2017).

### ﻿Diversity and composition of diatom assemblages

During the study 143 diatom taxa representing 39 genera were identified (Table 3, Figs 2, 3, 5–22). Most of them belonged to the genera *Luticola* (27), *Pinnularia* (13), and *Stauroneis* (10) (Table 3). Among the taxa identified, only a few species were found commonly in the materials studied and had noticeable shares in the communities. In most of the trees studied significantly more species were recorded in samples taken from the trunk bases (Fig. 4).

**Figure 5.**
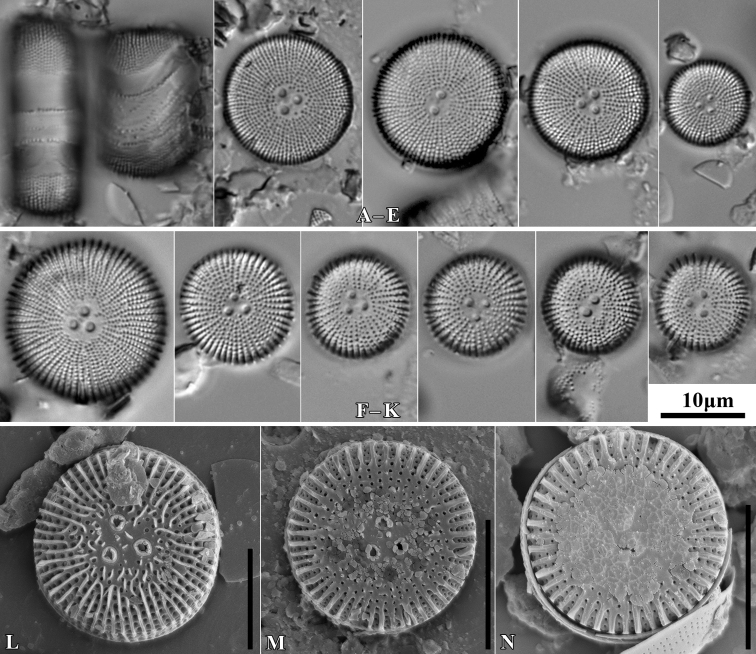
LM microphotographs of *Orthoseiradendroteres* in girdle view (**A**) and in size diminution series (**B–E**) and *O.roeseana* (**F–K**). SEM microphotographs of *Orthoseiradendroteres* (**L**) and *O.roeseana* (**M, N**). Scale bars: 10 µm.

**Figure 6.**
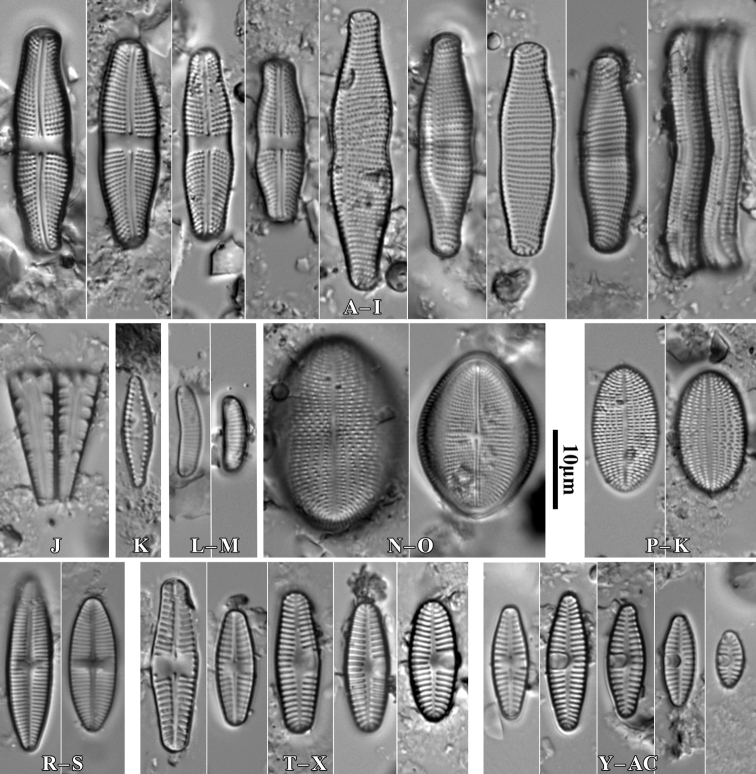
LM microphotographs of *Achnanthescoarctata* (**A–I**), *Meridioncirculare* (**J**), *Pseudostaurosirabrevistriata* (**K**), *Eunotiabotuliformis* (**L, M**), *Cocconeispediculus* (**N, O**), *C.lineata* (**P, K**), *Lemnicolahungarica* (**R, S**), *Planothidiumlanceolatum* (**T–X**) and *P.frequentissimum* (**Y–AC**). Scale bar: 10 µm.

**Figure 7.**
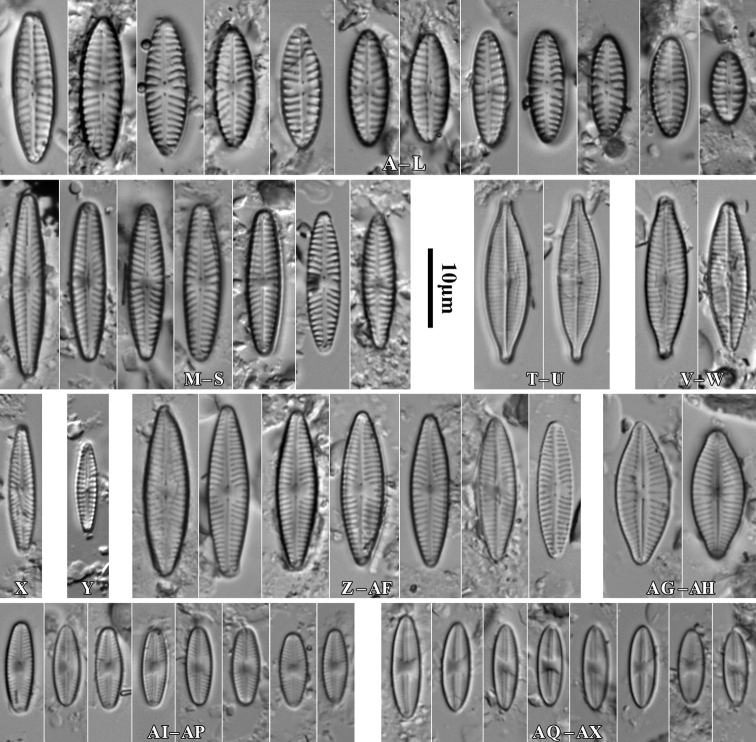
LM microphotographs of *Naviculaneowiesneri* (**A–L**), *N.pseudowiesneri* (**M–S**), *N.gregaria* (**T, U**), *N.veneta* (**V, W**), *N.tenelloides* (**X**), *N.bjoernoeyaensis* (**Y**), *N.lundii* (**Z–AF**), *Placoneishambergii* (**AG, AH**), *Sellaphorasubseminulum* (**AI–AP**) and *S.harderii* (**AQ–AX**). Scale bar: 10 µm.

**Figure 8.**
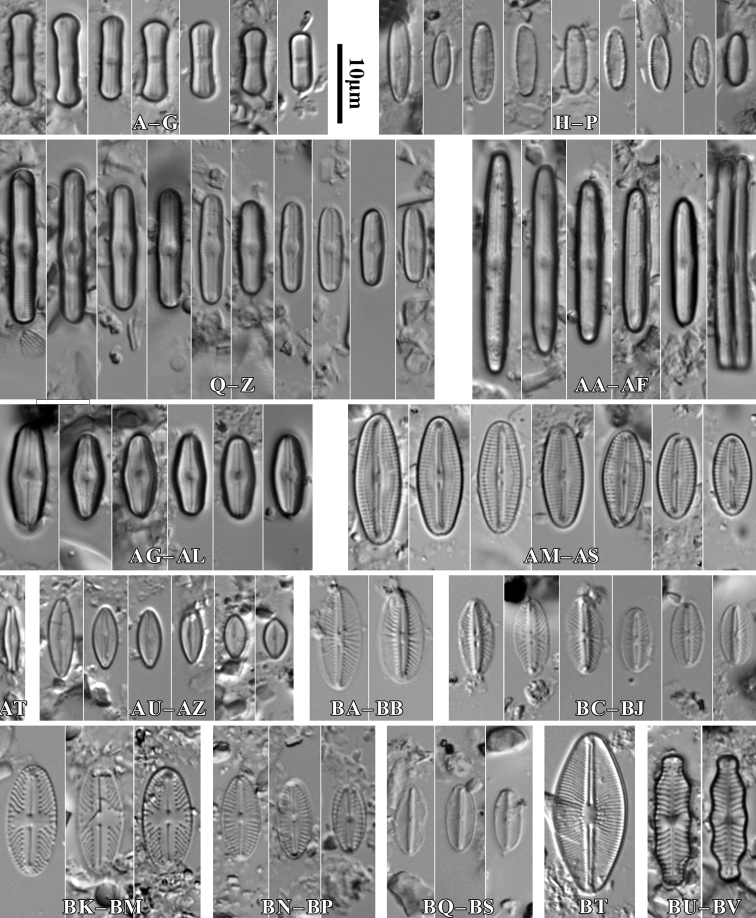
LM microphotographs of *Humidophilacontenta* (**A–G**), *H.gallica* (**H–P**), *H.brekkaensis* (**Q–Z**), *H.irata* (**AA–AF**), *H.perpusilla* (**AG–AL**), *Fallaciainsociabilis* (**AM–AS**), *F.enigmatica* (**AT**), *Microcostatusaerophilus* (**AU–AZ**), *Mayamaeaexcelsa* (**BA, BB**), *M.atomus* (**BC–BJ**), *M.asellus* (**BK–BM**), *M.fossalis* (**BN–BP**), *M.permitis* (**BQ–BS**), *Cavinulacocconeiformis* (**BT**) and *Geissleriaignota* (**BU, BV**). Scale bar: 10 µm.

**Figure 9.**
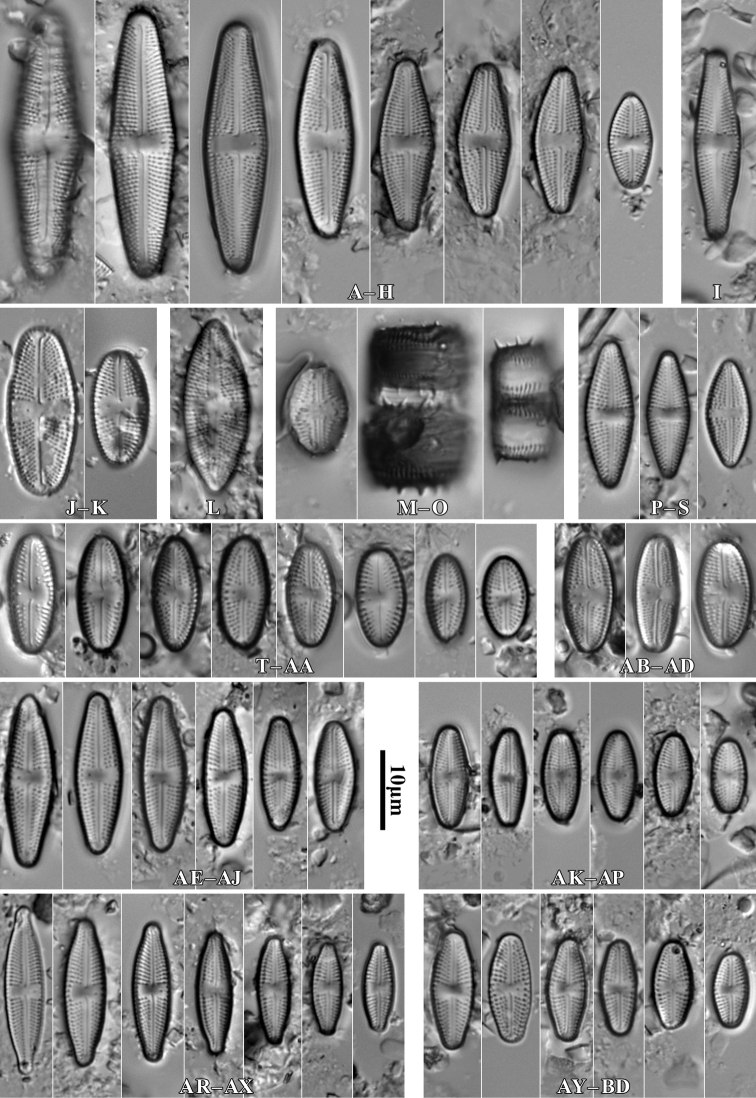
LM microphotographs of *Luticolaacidoclinata* (**A–H**), *L.poulickovae* (**I**), *L.cohnii* (**J, K**), *L.pseudokotschyi* (**L**), *L.spinifera* (**M–O**), *L.pitranensis* (**P–S**), *L.rotunda* (**T–AA**), *Luticola* sp. (**AB–AD**), *L.imbricata* (**AE–AJ**), *L.micra* (**AK–AP**), L.cf.vesnae (**AR–AX**) and *L.obscura* (**AY–BD**). Scale bar: 10 µm.

**Figure 10.**
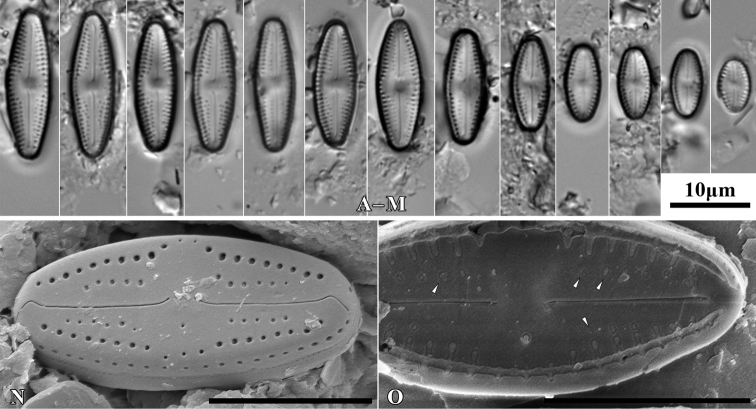
LM microphotographs of *Luticolasparsipunctata* in size diminution series (**A–M**). SEM microphotographs of external (**N**) and internal view (**O**). White arrowheads indicate a discontinuity in hymenes, Scale bars: 10 µm (**A–M**), 5 µm (**N, O**).

**Figure 11.**
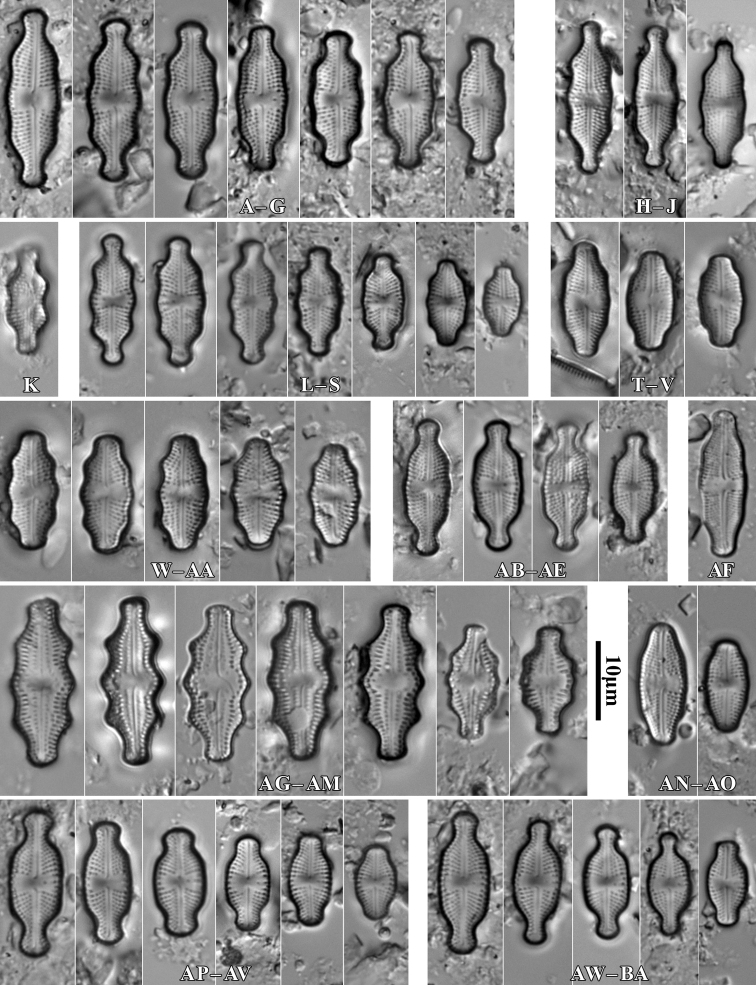
LM microphotographs of *Luticolanivalis* (**A–G**), *L.pseudonivalis* (**H–J**), *L.binodeformis* (**K**), *L.pulchra* (**L–S**), *L.kemalii* (**T–V**), *L.lecohui* (**W–AA**), *L.vanheurckii* (**AB–AE**), *L.undulata* (**AF**), *L.nivaloides* (**AG–AM**), *L.cholnokyi* (**AN, AO**), *L.ventricosa* MT1 (**AP–AV**) and *L.ventricosa* MT2 (**AW–BA**). Scale bar: 10 µm.

**Figure 12.**
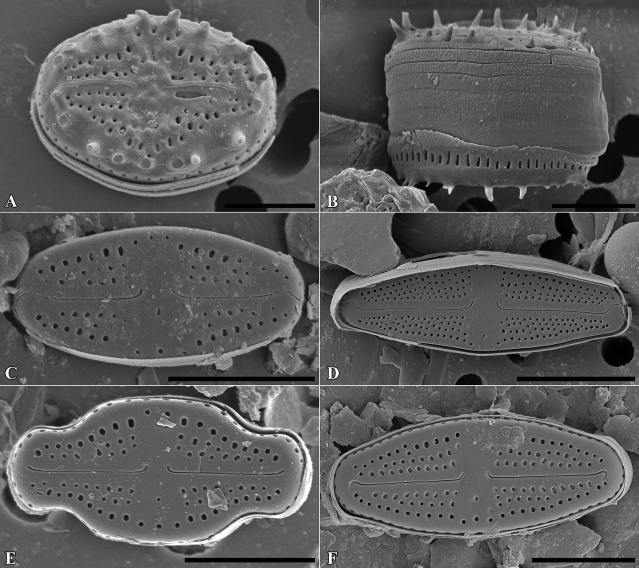
SEM microphotographs of *Luticolaspinifera* (**A, B**), *Luticola* sp. (**C**), *L.acidoclinata* (**D**), *L.ventricosa* MT2 (**E**) and *L.obscura* (**F**). Scale bars: 10 µm (**D**); 5 µm (**A–C, E, F**).

**Figure 13.**
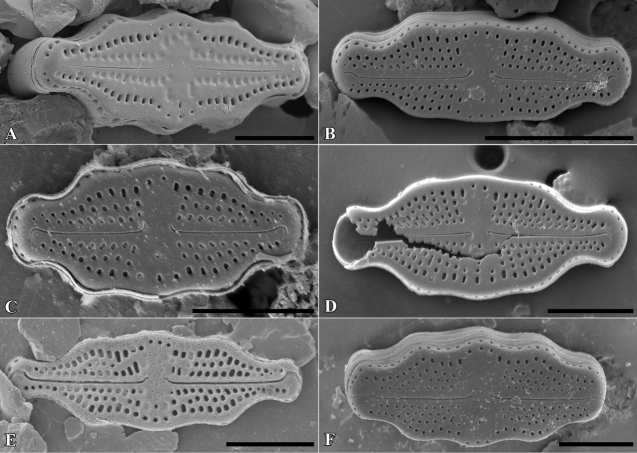
SEM microphotographs of *Luticolanivaloides* (**A**), *L.nivalis* (**B**), *L.pulchra* (**C**), *L.vanheurckii* (**D**), *L.pseudonivalis* (**E**) and *L.lecouhui* (**F**). Scale bars: 10 µm (**B**); 5 µm (**A, C–F**).

**Figure 14.**
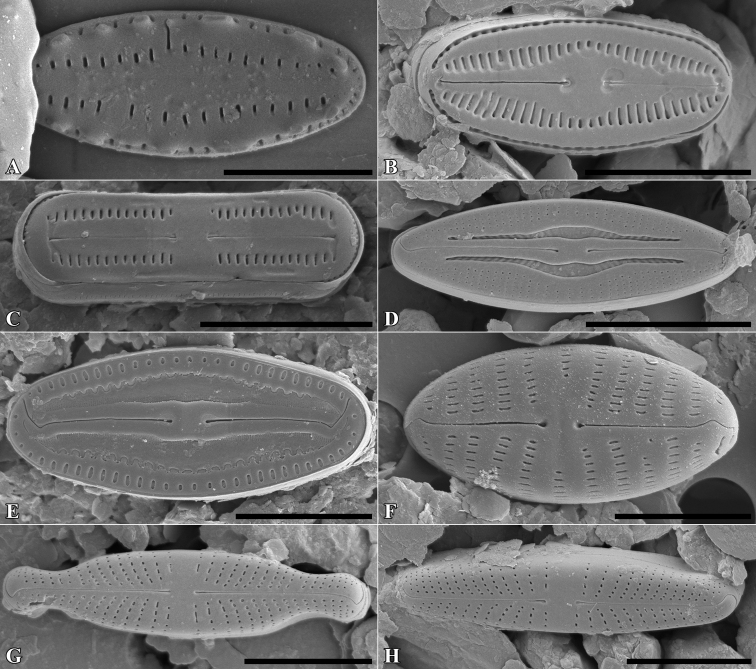
SEM microphotographs of *Humidophilagallica* (**A**), *H.perpusilla* (**B**), *H.contenta* (**C**), *Microcostatusaerophilus* (**D**), *Fallaciainsociabilis* (**E**), *Naviculaneowiesneri* (**F**), *Stauroneisthermicola* (**G**) and *S.parathermicola* (**H**). Scale bars: 5 µm (**B–H**); 3 µm (**A**).

**Figure 15.**
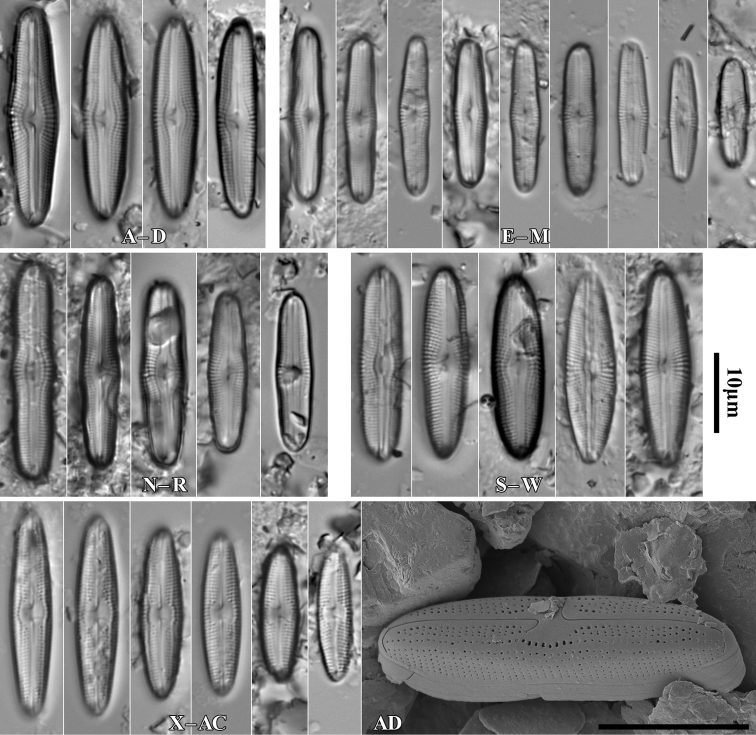
LM microphotographs of *Muelleriaislandica* (**A–D**), *M.undulata* (**E–M**), *M.terrestris* (**N–R**), *M.sasaensis* (**S–W**) and *Neidiumperforatum* (**X–AC**). SEM microphotography of *N.perforatum* (**AD**). Scale bar: 10 µm (**A–AC**); 5 µm (**AD**).

**Figure 16.**
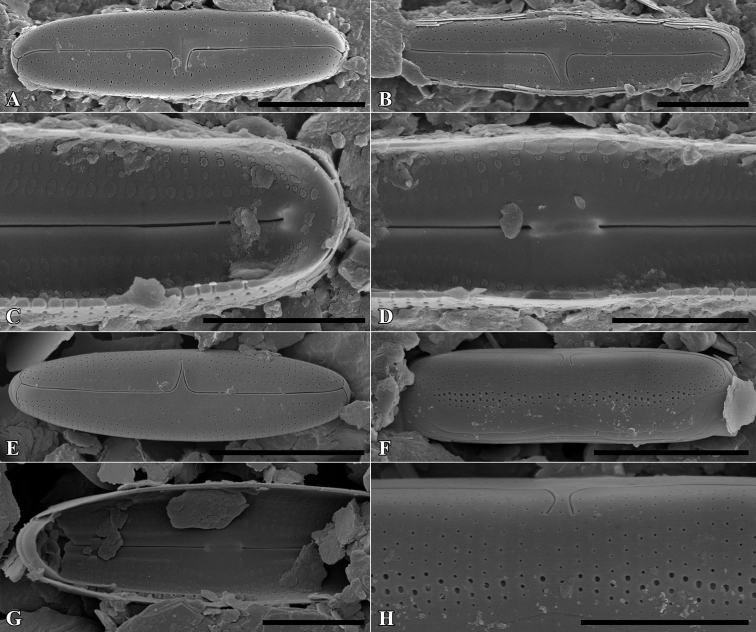
SEM microphotographs of *Muelleriaundulata* (**A–D**) and *M.terrestris* (**E–H**). Scale bars: 10 µm (**E, F**), 5 µm (**A, B, G, H**); 3 µm (**C, D**).

**Figure 17.**
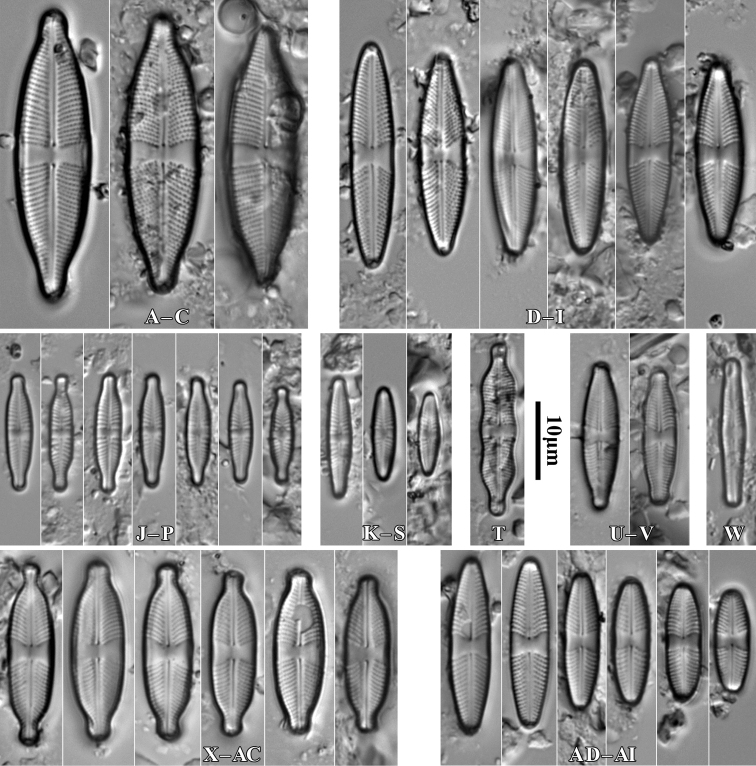
LM microphotographs of *Stauroneissaprophila* (**A–C**), *S.obtusa* (**D–I**), *S.thermicola* (**J–P**), *S.parathermicola* (**K–S**), *S.leguminopsis* (**T**), S.aff.lundii (**U, V**), *S.muriella* (**W**), *S.laterostrata* (**X–AC**), and *S.borichii* (**AD–AI**). Scale bar: 10 µm.

**Figure 18.**
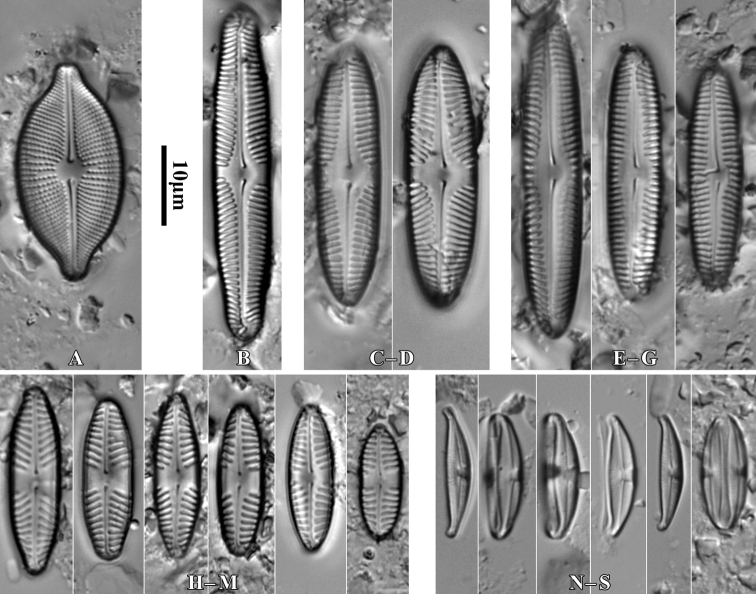
LM microphotographs of *Cosmioneispusilla* (**A**), Pinnulariamicrostauronvar.angusta (**B**), *P.brebissonii* (**C, D**), *P.isselana* (**E–G**), *P.cuneola* (**H–M**) and *Halamphoramontana* (**N–S**). Scale bar: 10 µm.

**Figure 19.**
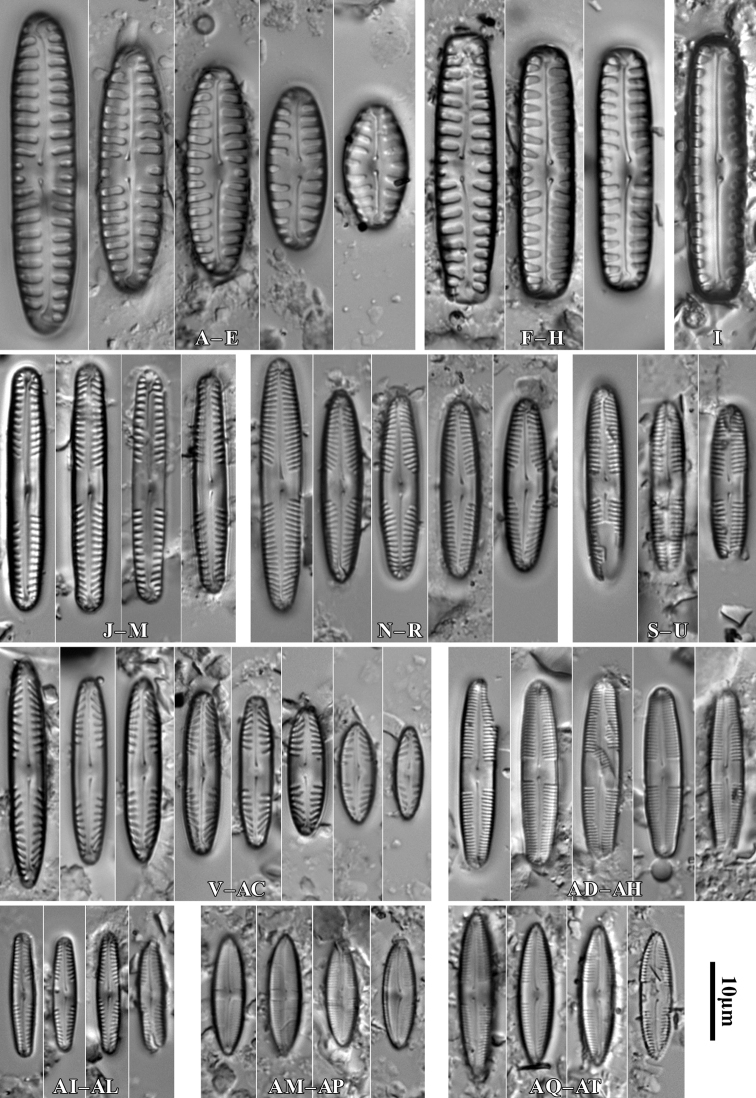
LM microphotographs of Pinnulariaborealisvar.borealis (**A–E**), P.borealisvar.subislandica (**F–H**), *P.dubitabilis* (**I**), *P.sinistra* (**J–M**), *P.schoenfelderi* (**N–R**), P.frauenbergianavar.caloneiopsis (**S–U**), *P.obscura* (**V–AC**), Caloneiscf.langebertalotioides (**AD–AH**), *P.perirrorata* (**AI–AL**), *C.vasileyevae* (**AM–AP**) and *C.lancettula* (**AQ–AT**). Scale bar: 10 µm.

**Figure 20.**
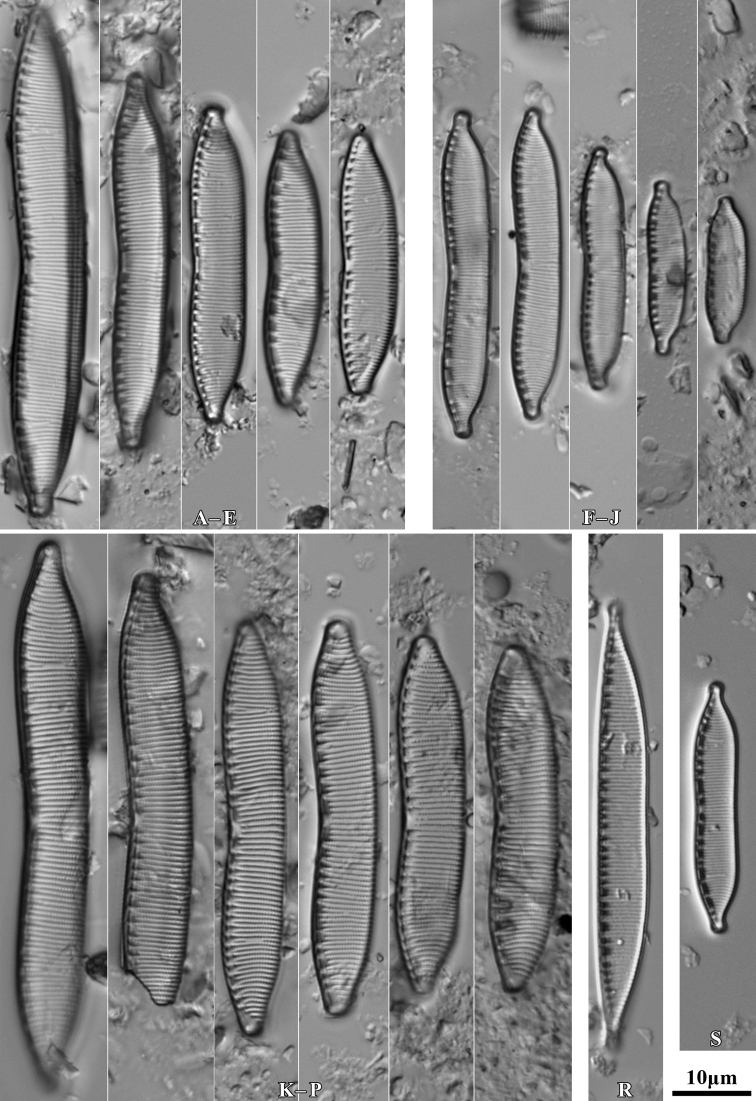
LM microphotographs of *Hantzschiaabundans* (**A–E**), *H.amphioxys* (**F–J**), *H.subrupestris* (**K–P**), *H.dorgaliensis* (**R**) and *H.stepposa* (**S**). Scale bar: 10 µm.

**Figure 21.**
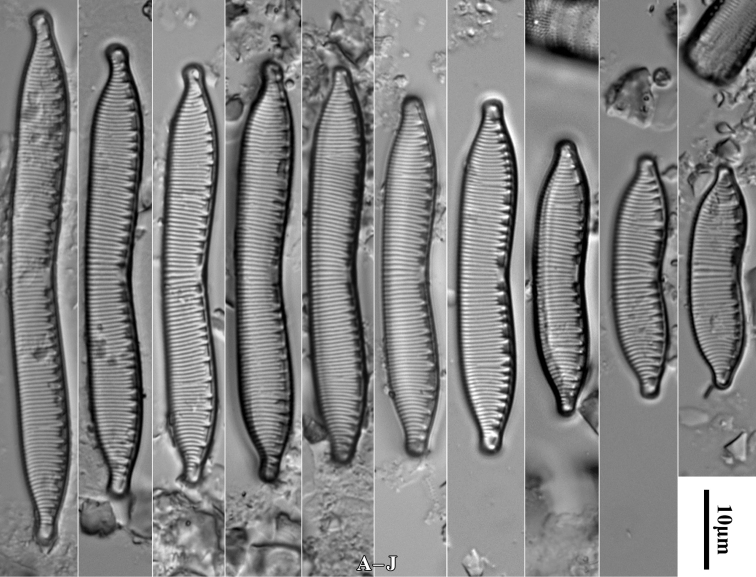
LM microphotographs of *Hantzschiacalcifuga* in size diminution series (**A–J**). Scale bar: 10 µm.

**Figure 22.**
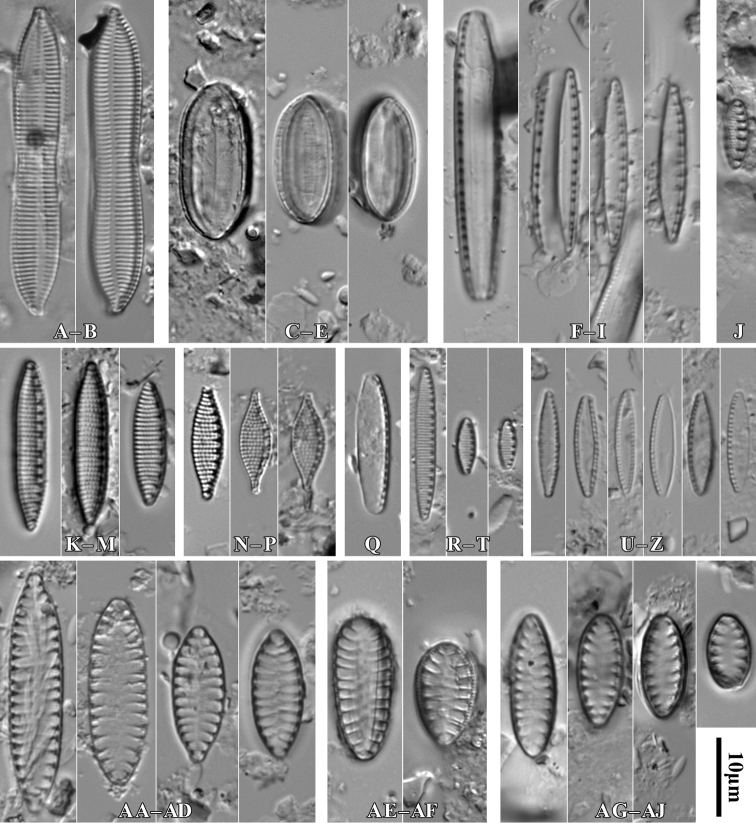
LM microphotographs of *Tryblionellaapiculata* (**A, B**), *T.debilis* (**C–E**), *Nitzschia harderi* (**F–I**), *N.solgensis* (**J**), *N.amphibia* (**K–M**), N.amphibiaf.apiculata (**N–P**), *N.communis* (**Q**), N.cf.frustulum (**R–T**), *N.pusilla* (**U–Z**), *Surirellaangusta* (**AA–AD**), *S.minuta* (**AE, AF**) and *S.terricola* (**AG–AJ**). Scale bar: 10 µm.

**Table 3.** Complete list of documented diatom taxa with measured dimension ranges (**Length / Width; striae/10µm**), frequency of occurrence in samples (**Freq.** [%]), minimum and maximum relative abundance (**Min-Max**) at sycamore maples (***Acer***), lindens (***Tilia***) and poplars (***Populus***). Bold indicates data from samples collected at 20 cm above ground level. + idicates observation of species in sample with unrepresentative assemblages.

Taxa	Dimensions	*Acer*		*Tilia*		*Populus*	
		Freq.	Min-Max	Freq.	Min-Max	Freq.	Min-Max
***Achnanthescoarctata*** (Brébisson & W.Smith) Grunow	15–32 / 4–7;	21	0.21–0.48	22	0.31–0.32	27	0.22–1.42
	11–13	**8**	**0.30–0.34**	**25**	**0.10–2.47**	**20**	**0.19**–**1.38**
***Achnanthidiumminutissimum*** (Kützing) Czarnecki	5–18 / 3;	11	0.24–0.48	4	+	8	–
	21–22	**9**	**0.21–0.31**	**12**	**0.32–7.41**	**15**	**0.19–0.49**
***Amphorapediculus*** (Kützing) Grunow	7–10 / 2.7–3;	–	–	–	–	–	–
	17–18	**1**	**0.32**	**5**	**0.41**	**1**	+
***Aulacoseira*** spp.	–	14	0.25–0.31	9	0.20	5	0.12–0.34
		**20**	**0.07–0.23**	**16**	**0.18–0.32**	**10**	**0.17–0.33**
***Caloneisaerophila*** W.Bock	12–19 / 3.5–4.5;			1	+		
	19–23			–	–		
***Caloneislancettula*** (Schulz) Lange-Bertalot & Witkowski	13–25 / 4–4.5;	4	0.37–0.48	1	+	2	–
	23–26	**2**	**0.17–0.21**	**3**	+	**3**	**0.65**
**Caloneiscf.langebertalotioides** Reichardt	17–26 / 4.5–5;	1	0.31	–	+	**1**	+
	26	–	**0.25**	**1**	**0.41**	**3**	**0.17**–**0.29**
***Caloneisleptosoma*** (Grunow) Krammer	23.5–32.5 / 5–6;	–	–	–	–		
	14–16	**1**	**0.26**	**1**	+		
***Caloneisvasileyevae*** Lange-Bertalot, Genkal & Vekhov	12–18 / 4–4.5;			1	+		
	27–30			–	–		
***Cavinulacocconeiformis*** (W.Gregory & Greville) D.G.Mann & A.J.Stickle	21–23.5 / 10;	1	0.30				
	27	–	–				
***Cocconeiseuglypta*** Ehrenberg	8–15 / 7–10;			1	+	1	+
	14–22			**6**	+	–	–
***Cocconeislineata*** Ehrenberg	8–15 / 8–11;	5	0.26–0.37	4	+	2	0.32
	14–26	**1**	**0.17**	**9**	**0.31**–**2.47**	**3**	**0.23**
***Cocconeispediculus*** Ehrenberg	17–26 / 8–11;			2	+	–	–
	18			**4**	**0.32**	**1**	**0.47**
***Cocconeispseudolineata*** (Geitler) Lange-Bertalot	7–20 / 7–11;	–	–	2	+	–	–
	15	**1**	**0.29**	**1**	+	**1**	**0.23**
***Cosmioneispusilla*** (W.Smith) D.G.Mann & A.J.Stickle	22.5–26 / 13–15;					1	+
	15–16					–	–
***Cyclostephanosdubius*** (Hustedt) Round	–	22	0.16–0.50	19	+	14	0.22–0.37
		**17**	**0.22–0.48**	**26**	**0.31**–**2.47**	**8**	**0.17–0.37**
***Epithemiaadnata*** (Kützing) Brébisson	17–22.5 / 7–9;	3	0.21–0.33	–	–	1	+
	14	**2**	**0.17–0.23**	**1**	**0.41**	**2**	**0.49**
***Eunotiabotuliformis*** F.Wild, Nörpel & Lange-Bertalot	6–26 / 3–3.5;	–	–	–	–		
	16–19	**8**	**0.21**–**0.37**	**1**	+		
***Eunotiaminor*** (Kützing) Grunow	12.5–31.5 / 4–6;			1	+	–	–
	10–12			–	–	**1**	**0.33**
***Fallaciaenigmatica*** (H.Germain) Lange-Bertalot & Werum	8.8 / 2–2.2;			–	–	2	–
	not visible in LM			**1**	+	–	+
***Fallaciainsociabilis*** (Krasske) D.G.Mann	6.5–21 / 4.5–6.5;	2	0.31–0.66	1	–	2	0.47
	22–25	**11**	**0.17–1.30**	**6**	**0.41**	**3**	**0.22–0.49**
***Geissleriaignota*** (Krasske) Lange-Bertalot & Metzeltin	22.5–24 / 5;					–	–
	13–14					**1**	**0.16**
***Gomphonellaolivacea*** (Hornemann) Rabenhorst	12–23 / 5–6;	1	0.32	–	–	–	–
	10–11	–	–	**1**	+	**1**	+
***Gomphonemaacuminatum*** Ehrenberg	22 / 7;	1	0.24	–	–		
	12	–	–	**1**	+		
***Gomphonemaamoenum*** Lange-Bertalot	30–35 / 9–10;	1	0.31			–	–
	10–11	**1**	**0.32**			**1**	**0.24**
***Gomphonemadrutelingense*** Reichardt	20–24.5 / 6.5–7;					–	–
	11					**1**	+
***Gomphonemamicropus*** Kützing	20–26 / 7–7.5;	2	0.13	1	+	1	+
	10–12	**4**	**0.26–0.29**	**7**	**0.20–1.23**	**4**	**0.32**–**0.93**
***Gomphonemaparvulum*** (Kützing) Kützing	10–15 / 6–7;	1	0.24–	–	–	2	–
	11–13	–		**2**	+	**1**	**0.31**
***Halamphoramontana*** (Krasske) Levkov	12–20 / 3.5–5.5;	18	0.20–0.34	13	0.31–0.32	16	0.17–3.79
	not visible in LM	**21**	**0.17–2.08**	1**6**	**0.10–0.41**	**16**	**0.17–0.65**
***Hantzschiaabundans*** Lange-Bertalot	35–74.5 / 6–9.5;	47	0.21–2.81	51	0.30–0.61	51	0.12–6.16
	19–20	**43**	**0.17**–**9.95**	**77**	**0.20–2.40**	**65**	**0.16–6.92**
***Hantzschiaamphioxys*** (Ehrenberg) Grunow	12–45 / 4.5–7;	88	0.20–23.13	84	0.30–2.76	87	0.12–93.90
	21–27	**94**	**0.14–58.82**	**99**	**0.20–29.94**	**91**	**0.16–88.10**
***Hantzschiacalcifuga*** Reichardt & Lange-Bertalot	28–85 / 6.5–7.7;	18	0.21–23.18	20	+	18	0.17–0.47
	16–19	**37**	**0.18–4.02**	**25**	**0.18**–**4.12**	**22**	**0.22–21.80**
***Hantzschiadorgaliensis*** Lange-Bertalot, Cavacini, Tagliaventi & Alfinito	60 / 6.7;					–	–
	18–19					**1**	+
**Hantzschiaaff.stepposa** Maltsev & Kulikovskiy	33 / 5;					–	–
	25					**1**	+
***Hantzschiasubrupestris*** Lange-Bertalot	26.7–81 / 6.7–9;	9	0.25–0.66	10	+	13	0.35–0.95
	14–17	**15**	**0.18–2.99**	**16**	**0.20**–**0.65**	**20**	**0.16–1.01**
***Humidophilabrekkaensis*** (Petersen) Lowe et al.	8–19.5 / 6–6.5;	1	–	1	+	2	–
	not visible in LM	**5**	**0.24**–**12.5**	**5**	+	**3**	**0.34**
***Humidophilacontenta*** (Grunow) Lowe et al.	5–13 / 2.5–3.2;	53	0.20–23.13	32	7.50–58.51	40	0.56–59.25
	not visible in LM	**68**	**0.02–34.05**	**52**	**0.30–79.22**	**72**	**0.23–81.77**
***Humidophilagallica*** (W.Smith) Lowe et al.	4–10 / 2–3;	2	0.31	9	0.20	10	0.35–10.90
	not visible in LM	**3**	**0.22–0.34**	**7**	+	**15**	**0.22–4.12**
***Humidophilairata*** (Krasske) Lowe et al.	15–21 / 6.2–6.5;	6	0.24–0.31	2	+	2	0.24
	not visible in LM	**25**	**0.17–1.01**	**17**	**0.10–0.41**	**6**	**0.19**–**0.22**
***Humidophilaperpusilla*** (Grunow) Lowe et al.	6–13.5 / 4–5;			1	+	5	0.84
	not visible in LM			**2**	+	**10**	**2.21–19.06**
***Lemnicolahungarica*** (Grunow) Round & Basson	8–13 / 4–6;	–	–	1	+	–	–
	18–21	**1**	**0.25**–**0.34**	**2**	+	**1**	**0.49**
***Luticolaacidoclinata*** Lange-Bertalot	10–32 / 4.8–8.7;	81	0.31–98.77	50	2.50–38.21	55	1.02–97.33
	18–23	**87**	**0.26–99.91**	**69**	**1.62–94.12**	**68**	**7.73–91.22**
***Luticolabinodeformis*** Levkov, Metzeltin & Pavlov	12–15.5 / 4–4.5;					–	–
	21–22					**1**	**0.33**
***Luticolablancoi*** Levkov, Tofilovska, C.E.Wetzel, Mitić-Kopanja & Ector	18–21 / 6.5;			–	–	–	–
	20–21			**1**	+	**1**	+
***Luticolabryophila*** M.Rybak, Czarnota & Noga, sp. nov.	10–25 / 4–6;	5	0.26	8	+	5	0.24
	18–20	**14**	**0.17**–**0.66**	**11**	**0.25**–**0.65**	**17**	**0.22**–**7.73**
***Luticolacholnokyi*** Levkov, Metzeltin & Pavlov	15–18 / 6.5;	–	–				
	20–21	**1**	0.16				
***Luticolacohnii*** (Hilse) D.G.Mann	17.5–28.5 / 8.5–10;	–	–	–	–		
	20–21	**1**	0.31	**1**	+		
***Luticolaconfusa*** M.Rybak & Czarnota, sp. nov.	9–22 / 4.5–6;	20	0.24–0.32	13	–	20	0.35–3.32
	20–22	**25**	**0.18–10.45**		**0.18-2.00**	**19**	**0.17–0.53**
***Luticolaimbricata*** (W.Bock) Levkov, Metzeltin & Pavlov	15–17 / 5.5–7;	–	–	1	+	–	–
	20	**2**	**0.30**–**0.66**	**1**	+	**1**	+
***Luticolakemalii*** Solak & Levkov	12–16 / 5.6–7;	2		–	–	2	+
	20–22	–		**1**	+	**2**	**0.28**
***Luticolalecohui*** Levkov, Tofilovska, C.E.Wetzel, Mitic-Kopanja & Ector	15–23.5 / 7–8;	1	+	1	+		
	20–21	–	–	**1**	+		
***Luticolamicra*** Levkov, Metzeltin & Pavlov	7.5–13 / 3.8–4.5;	6	0.24–0.32	5	0.30–0.31	7	0.24–0.28
	22–24	**9**	**0.20–0.52**	**5**	**0.28–0.30**	**2**	**0.19**
***Luticolanivalis*** (Ehrenberg) D.G.Mann	10–21 / 5.8–7.5;	16	0.28–0.36	15	0.32	21	0.17–0.95
	19–21	**17**	**0.25–0.68**	**9**	**0.10**	**33**	**0.22–0.69**
***Luticolanivaloides*** (W.Bock) Denys & De Smet	15–23.5 / 6.5–8;	1	0.48	1	+	6	0.47
	18–19	–	–	**4**	**0.32**	**8**	**0.17–0.29**
***Luticolaobscura*** Levkov, Tofilovska, C.E.Wetzel, Mitić-Kopanja & Ector	10–24.5 / 5–7;	20	0.48–0.48	28	0.30–0.31	15	0.34
	19–22	**26**	**0.17–2.24**	**36**	**0.18–1.53**	**29**	**0.19–7.54**
***Luticolapitranensis*** Levkov, Metzeltin & Pavlov	15–22.5 / 5.5–6.5;	–	–				
	20–22	**2**	**0.17**–**0.20**				
***Luticolapoulickovae*** Levkov, Metzeltin & Pavlov	22 / 6;	–	–				
	21	**1**	+				
***Luticolapulchra*** (McCall) Levkov, Metzeltin & Pavlov	10–20 / 5.9–6.4;	1	0.32	7	0.31	15	1.90
	20–24	**6**	**0.22–0.34**	**9**	**0.25–0.30**	**13**	**0.19–0.33**
***Luticolapseudonivalis*** (W.Bock) Levkov, Metzeltin & Pavlov	13.2–16.5 / 5–6;	4	+	2	0.31	1	0.47
	24	**1**	**0.28**	–	–	–	–
***Luticolarotunda*** Solak & Levkov	12–15.5 / 6–6.5;	2	0.20–0.31	1	+	2	–
	19–20	**8**	**0.31–0.68**	**1**	+	**1**	**0.22**–**0.29**
***Luticolasparsipunctata*** Levkov, Metzeltin & Pavlov	9.5–27 / 4.5–7;	14	0.28–0.40	19	0.31	**25**	0.12–1.42
	18–20	**24**	**0.17–3.67**	**27**	**0.18–0.34**	**32**	**0.19–7.80**
***Luticolaspinifera*** (W.Bock) L.Denys & W.H.De Smet	12–14 / 7–8;					1	+
	16					–	–
***Luticolaundulata*** (Hilse) D.G.Mann	19–22.5 / 7.5–7.8;			–	–	–	–
	24–25			**1**	+	**1**	**0.49**
***Luticolavanheurckii*** Van de Vijver & Levkov	14.5–19 / 5.5–7.3;			10	+	6	+
	18–21			**16**	**0.25**–**0.65**	**3**	**0.22**–**0.29**
***Luticolaventricosa*** (Kützing) D.G.Mann **MT1**	10–23 / 5.5–7;	20	0.23–0.40	16	0.30–0.31	15	0.24–3.79
	19–22	**11**	**0.22–10.95**	**17**	**0.25–0.32**	**27**	**0.17–2.07**
***Luticolaventricosa*** (Kützing) D.G.Mann **MT2**	9.2–22 / 5–7;	10	0.26–0.61	23	0.30–0.31	35	0.12–1.42
	19–22	**13**	**0.02–0.82**	**43**	**0.19–62.21**	**31**	**0.19–0.69**
***Luticola*** cf. ***vesnae*** Levkov, Metzeltin & Pavlov	10–23.7 / 5–7.2;	8	0.16–0.25	7	+	9	0.12–0.34
	20–24	**24**	**0.18–7.12**	**18**	**0.18**–**0.32**	**13**	**0.28–0.97**
***Luticola*** sp. 1	14–18 / 5.5–7;	–	–	1	0.31	2	0.25
	18–23	**9**	**0.34**–**0.37**	**9**	**0.32–0.41**	**5**	**0.16–0.19**
***Luticola*** sp. 2	-	–	+	–	–	–**1**	+
		**1**	**0.28**	**1**	+	**1**	**0.28**
***Mayamaeaasellus*** (Weinhold & Hustedt) Lange-Bertalot	12–16.5 / 5.5–6;	1	0.32	2	+	4	+
	16–18	**4**	+	**7**	**0.25**–**0.32**	**2**	**0.28**–**0.29**
***Mayamaeaatomus*** (Kützing) Lange-Bertalot	7–13 / 4–5;	5	+	9	0.31	7	0.22–0.95
	18–22	**23**	**0.17**–**6.86**	**37**	**0.25–0.41**	**15**	**0.17–0.49**
***Mayamaeaexcelsa*** (Krasske) Lange-Bertalot	11–14.5 / 5–7;	2	0.22	–	–	1	+
	16–17	**5**	**0.26–0.31**	**7**	+	**3**	**0.26**–**0.33**
**Mayamaeafossalisvar.fossalis** (Krasske) Lange-Bertalot	9–11.5 / 3–4;	1	+	1	+	1	+
	17–19	**3**	**0.17**–**0.41**	**4**	+	**1**	**0.33**
**Mayamaeafossalisvar.obsidialis** (Hustedt) Lange-Bertalot	9–11 / 4.5;			–	–		
	18			**3**	**0.18**		
***Mayamaeapermitis*** (Hustedt) K.Bruder & Medlin	7–12 / 3.5–4.5;	1	+	1**11**	1	2	+
	ca. 35	**7**	**0.17**–**2.69**	**11**	**0.20**	**2**	**0.19**
***Meridioncirculare*** (Greville) C.Agardh	13–24 / 5–6	3	0.20–0.31	7	+	2	0.24
		**2**	+	**9**	**0.41**	**1**	+
***Microcostatusaerophilus*** Stanek-Tarkowska, Noga, C.E.Wetzel & Ector	6–9.5 / 2.5–3.5;	1	+	–	–		
	not visible in LM	–	–	**5**	+		
***Muelleriaislandica*** (Østrup) Lange-Bertalot	19.5–30 / 6.7–7.7;	2	+	2	+	3	+
	22–25	**5**	**0.17–0.35**	**3**	+	**2**	+
***Muelleriasasaensis*** Levkov, Vidaković, Cvetkoska, Mitić-Kopanja, Krstić, Van de Vijver & Hamilton	22.5–29.5 / 5.8–6.2;	–	–	3	+	1	–
	23–24	**3**	**0.24**–**1.49**	**1**	+	**3**	**0.25**
***Muelleriaterrestris*** (J.B.Petersen) Spaulding & Stoermer	23.5–26 / 5.2–6;	3	0.24	2	+	3	+
	18–19	**3**	**0.23–0.64**	**3**	**0.50**	**3**	v
***Muelleriaundulata*** (Krasske) Levkov, Hamilton & Van de Vijver	12–22 / 4.3–5;	3	+	1	+	–	–
	26–28	**3**	**0.17**–**3.98**	–	–	**2**	+
***Naviculabjoernoeyaensis*** Metzeltin, Witkowski & Lange-Bertalot	17–18 / 3;	1	+	–	–	1	0.34
	18	–	–	**1**	+	**3**	**0.23**
***Naviculagregaria*** Donkin	15.5–22 / 6–7;	–	–	1	+	1	0.12
	16	1	**0.31**	**1**	**0.41**	**3**	**0.29–0.43**
***Naviculalundii*** Reichardt	14–25 / 4.2–6;	–	–			1**1**	0.47
	15	**2**	**0.24**–**0.50**				**0.24**
***Naviculaneowiesneri*** Chaudev & Kulikovskyi	12–32 / 4.5–6;	38	0.16–0.80	36	0.20–0.32	43	0.12–5.69
	11–13	**35**	**0.18–2.03**	40	**0.18–1.75**	38	**0.17–4.14**
***Naviculapseudowiesneri*** Chaudev & Kulikovskyi	11–24 / 4–5;	30	0.16–0.62	21	0.18–0.24	22	0.12–1.12
	11–13	**35**	**0.18–2.03**	**23**	**0.18–1.02**	**12**	**0.17–0.35**
***Naviculatenelloides*** Hustedt	14–18 / 3–3.5;	–	–	1	+	1	+
	16	**1**	**0.25**	**1**	**0.41**	–	–
***Naviculaveneta*** Kützing	12–25.5 / 4.5–5.5;	1	+	–	–	1	0.12
	13–16	**2**	**0.32**–**0.41**	**2**	**0.32**	**4**	**0.22–0.49**
***Neidiumalpinum*** Hustedt	15–29 / 4.5–5;			–	–		
	ca. 37			**1**	+		
***Neidiumperforatum*** Schimanski	15.5–26 / 4.5–5.5;	2	0.31–0.33				
	21–23	**6**	**0.18–0.37**				
***Nitzschiaamphibia*** Grunow	9–28 / 4–5.5;	9	0.21–0.48	6	0.31	8	0.34–0.47
	15–18	**7**	**0.25–0.41**	**7**	**0.32**	**8**	**0.24–0.49**
**Nitzschiaamphibiaf.rostrata** Hustedt	12–15 / 3.5–4;	1		–	–	–	–
	18–20	–		**1**	**0.32**	**1**	+
***Nitzschiacommunis*** Rabenhorst	15–20 / 4;	1	0.24			1	+
	not visible in LM	–	–			**3**	**0.22**–**0.29**
***Nitzschiaharderi*** Hustedt	19–36 / 3–4;	1	0.25				
	not visible in LM	**1**	**1.48**				
***Nitzschiapusilla*** Grunow	7–22 / 2.5–3.5;	5	0.21–0.36	5	0.20	2	+
	not visible in LM	**11**	**0.18–1.59**	**5**	**0.10**	**7**	**0.22**–**0.33**
**Nitzschiacf.frustulum** (Kützing) Grunow	4–16.5 / 3;	15	0.25–0.36	9	–	4	0.12
	25–27	**6**	**0.30**	**16**	**0.32**–**0.34**	**14**	**0.22–6.45**
***Nitzschiasolgensis*** Cleve-Euler	11.5–24 / 3–4;	1	0.24	–	–	3	0.33
	21–23	**2**	**0.30**	**1**	+	**3**	**0.19–0.29**
**Nitzschiacf.supralitorea** Lange-Bertalot	10–20 / 2.5–3.5;			–	–	2	1.42
	27–30			**1**	+	–	–
***Nitzschialinearis*** W.Smith	50–55 / 6;					–	–
	28–29					2	**0.22**–**1.74**
***Orthoseiradendroteres*** (Ehrenberg) Genkal & Kulikovskiy	Ø – 7–26;	36	0.24–95.69	7	+	9	0.17–3.59
	20–22	**39**	**0.23–95.36**	**17**	**0.82**	16	**0.17–72.02**
***Orthoseiraroeseana*** (Rabenhorst) Pfitzer	Ø – 8–24;	4	0.24–0.71	2	+	1	0.17
	8–12	**8**	**0.17–3.55**	–	–	**4**	**0.2**6–**2.85**
**Pantocsekiellacf.ocellata** (Pantocsek) K.T.Kiss & E.Ács	–	6	0.24–0.34	2	+	6	0.12–0.33
		**10**	**0.02–0.20**	**6**	+	**11**	**0.19–0.33**
**Pinnulariaborealisvar.borealis**Ehrenberg	22–42 / 8–9.5;	68	0.21–58.21	52	0.30–1.81	57	1.63–97.80
	ca. 5	**80**	**0.03–52.24**	**79**	**0.30–86.57**	**67**	**0.24–66.32**
**Pinnulariaborealisvar.subislandica** Krammer	35–40 / 8.5–9;	–	–	1	+	1	0.47
	ca. 5	**3**	**0.26**–**0.32**	**2**	**0.30**	**3**	**0.29–0.65**
***Pinnulariabrebissonii*** (Kützing) Rabenhorst	10–34 / 9–12;	1	+	1	+	2	+
	10–13	**3**	**0.22–0.34**	**1**	+	**2**	**0.22**–**0.29**
***Pinnulariacuneola*** Reichardt	24.5–30 / 7–8;	9	0.28–0.34	3	+	9	0.34
	10–11	**7**	**0.23–0.32**	**11**	**0.30**–**0.41**	**9**	**0.17–0.49**
***Pinnulariadubitabilis*** Hustedt	40 / 9;	–	–				
	ca. 5	**1**	+				
**Pinnulariaaff.frauenbergianavar.caloneiopsis** Lange-Bertalot & M.Werum	13–22 / 4–4.5;	3	+	1	+	2	+
	17–18	**2**	**0.33**–**1.35**	**2**	+	**1**	**0.29**
***Pinnulariaisselana*** Krammer	30–45 / 7–9;	2	0.28–0.36	2	+	1	0.24–0.47
	10–12	–	–	**10**	**0.32**–**0.41**	**5**	**0.24–0.49**
**Pinnulariamicrostauronvar.angusta** Krammer	42–44 / 6.5–7;			1	+		
	12–13			–	–		
**Pinnulariamicrostauronvar.rostrata** Krammer	35 / 6.5;			1	+		
	11			–	–		
***Pinnulariaobscura*** Krasske	9–35 / 3–5;	55**59**	0.21–1.95	29	0.20–0.31	3	0.24–1.90
	10–13		**0.02–26.48**	**73**	**0.18–9.48**	**38**	**0.16–2.03**
***Pinnulariaperirrorata*** Krammer	12–25 / 4;	–	–	1	+		
	16–18	**5**	**0.25**–**0.27**	**7**	**0.30**		
***Pinnulariaschoenfelderi*** Krammer	19–34 / 5–6.5;	10	0.22–0.50	11	+	6	0.33
	14–16	**11**	**0.22–0.37**	**20**	**0.32**–**2.47**	**7**	**0.23–0.32**
***Pinnulariasinistra*** Krammer	17–35 / 4.5–6;	3	0.21–0.36	1	+	2	+
	11–13	**6**	**0.27–0.37**	**8**	**0.18**	**2**	+
***Placoneishambergii*** (Hustedt) K.Bruder	14–22 / 5–7;	1	+	–	–		
	13–16	**4**	**0.26**–**0.30**	**3**	**0.41**		
***Planothidiumfrequentissimum*** (Lange-Bertalot) Lange-Bertalot	4–25 / 3.5–6;	6	0.16–0.36	5	0.31	8	0.22–2.37
	14–17	**5**	**0.21–0.30**	**22**	**0.30–2.47**	**11**	**0.19–1.69**
***Planothidiumlanceolatum*** (Brébisson & Kützing) Lange-Bertalot	7–25 / 4–6.5;	5	0.21–0.30	5	0.30	2	0.12
	13–15	**3**	**0.29–0.30**	**8**	**0.30–3.29**	**7**	**0.22–3.72**
***Rhoicospheniaabbreviata*** (C.Agardh) Lange-Bertalot	–			–	–	–	–
				**1**	+	**1**	+
***Reimeriasinuata*** (W.Gregory) Kociolek & Stoermer	9–14 / 3.5–5.5;	–	–	–	–		
	9–13	**1**	**0.29**	**1**	+		
***Sellaphoraatomoides*** (Grunow) Wetzel & Van de Vijver	6–12 / 3–4;	5	0.31–0.36	7	+	4	0.12–1.42
	22–25	**8**	**0.17–0.41**	**14**	**0.41**	8	**0.12–0.33**
***Sellaphoraharderi*** (Hustedt) J.Foets & C.E.Wetzel	5.5–12 / 3–4;	–	–	–	–	1	+
	32–35	**1**	**0.28**	**2**	+	–	–
***Sellaphoranana*** (Hustedt) Lange-Bertalot, Cavacini, Tagliaventi & Alfinito	15–18.5 / 4–4.5;	2	0.30	–	–	1	0.47
	38	**1**	**0.41**	**3**	+	–	–
***Sellaphoranigri*** (De Notaris) C.E.Wetzel & Ector	5–10 / 4;					1	0.47
	28–30					**1**	+
***Sellaphorasubseminulum*** (Hustedt) Wetzel	7–14 / 3.2–3.8;	1	+	2	0.31	1	+
	23–25	**8**	**0.19**–**1.23**	**15**	**0.41–3.29**	**5**	**0.22**–**0.34**
***Stauroneisborrichii*** (J.B.Petersen) J.W.G.Lund	10–26 / 3.8–5;	19	0.22–0.33	22	0.20	18	0.17–0.95
	22–26	**40**	**0.20–9.97**	**43**	**0.10–1.19**	**26**	**0.19–0.49**
***Stauroneislaterostrata*** Hustedt	19–26 / 6.5–8.5;	–	–			5	+
	17–20	**8**	**0.17**–**2.01**			**5**	+
***Stauroneisleguminopsis*** Lange-Bertalot & Krammer	20–22 / 4.5;	–	–				
	24	**1**	**0.31**				
***Stauroneis*** aff. ***lundii*** Hustedt	14–19 / 4–5;	2	0.20	4	+	4	0.17–0.22
	23–24	**16**	**0.22–0.59**	**12**	**0.32**–**1.03**	**4**	**0.22–0.29**
***Stauroneismuriella***J.W.G.Lund	16–20 / 3–4;	1	+	2		–	–
	22–26	**2**	**0.26**	**1**		**1**	+
***Stauroneisobtusa*** Lagerstedt	15–28 / 6–8;	1	+	3	+	2	+
	19–23	**4**	**0.30**–**6.25**	**5**	**0.41**	**2**	+
***Stauroneisparathermicola*** Lange-Bertalot	8–18 / 3–4.5;	2	0.32–0.34	1	+	2	+
	20–23	**10**	**0.17–1.01**	**12**	**0.82**	**2**	**0.22**–**0.24**
***Stauroneissaprophila*** M.Rybak, Noga & Ector	30–35/ 8.5–9.5;	3	0.48	1	+	3	+
	15–17	2	**0.48**	**2**	+	**4**	**0.22**–**0.49**
***Stauroneisseparanda*** Lange-Bertalot & Werum	13–14 / 3.8–4.2;	1	0.30	1	+		
	ca. 28	**1**	**0.30**	**1**	+		
***Stauroneisthermicola*** (J.B.Petersen) J.W.G.Lund	8–18 / 3–4.5;	18	0.21–0.37	7	+	2	0.12
	20–23	**28**	**0.18–4.39**	**31**	**0.10**–**0.32**		**0.22–0.33**
***Surirellaangusta*** Kützing	16.5–42.5 / 6–9;	3	+	1	+	2	0.12–0.47
	24–27	–	–	**2**	**0.30**	**7**	**0.16–1.16**
***Surirellaminuta*** Brébisson & Kützing	9–34.5 / 8–10;	–	–	2	+	2	+
	26–28	**1**	**0.25**	**6**	**0.41**	**3**	**0.29**–**0.23**
***Surirellaterricola*** Lange-Bertalot & E.Alles	11–18.1 / 6.4;	10	0.21–0.36	9**7**	+	2	+
	18–23	**10**	**0.23–0.34**		**0.41**	**3**	**0.28**–**0.32**
***Tryblionellaapiculata*** W.Gregory	20–24 / 4.5–5.5;					–	–
	17					**2**	**0.49**–**1.08**
***Tryblionelladebilis*** Arnott & O’Meara	12–23.5 / 7–8.5;	–	–	1**1**	+	–	–
	not visible in LM	**1**	+		+	**3**	**0.22**–**0.49**
***Tryblionellahungarica*** (Grunow) Frenguelli	35–42 / 5.2–6;			–	–	–	–
	8–10			**1**	**0.25**	**2**	**0.22**–**0.29**

Of the 647 samples collected, only in 197 were numerous occurrences of diatoms observed. Diatoms did not occur, or occurred only, as individual valves in all samples from barks covered with lichens or algal mats. Numerous diatom assemblages were observed in 74 of 283 samples from bare bark (27 from 20 cm above ground level and 47 from 150 cm above ground level). Numerous assemblages were also observed in 123 of 231 moss samples collected (43 from 20 cm above ground level and 80 from 150 cm above ground level).

Higher values of both indices studied (H’ and J’) were usually recorded for bare bark samples, and higher values of both of these indices were also recorded for samples collected from trunk bases (Fig. 4). When more than one microhabitat from a single tree was analyzed in the same season, higher values of both indexes were noted for bare bark (Fig. 4).

**Figure 23.**
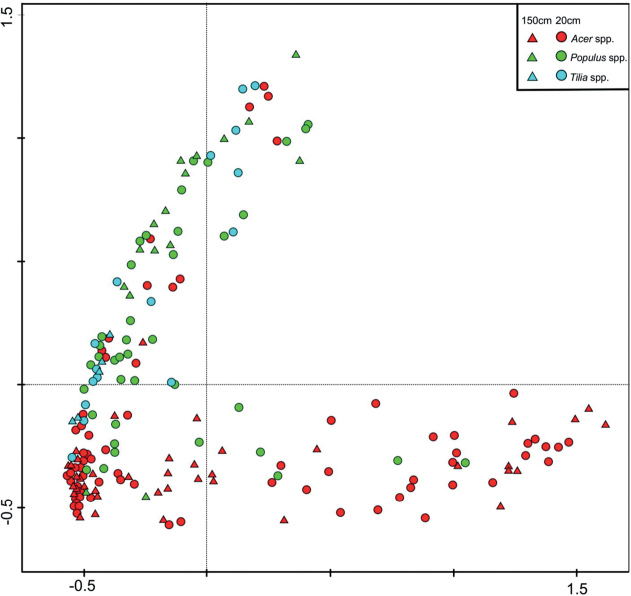
PCA ordination of analyzed samples determined by relative diatom assemblage composition on studied tree taxa.

Principal component analysis (PCA) revealed considerable variability in the diatom assemblages. The gradient length in analysis was 2.7. The first ordination axis explained 33.48% of the variation, the second 25.00%, the third 14.02%, and the fourth 10.14% (Fig. 23). The diatom communities from sites located in city centers and small peripheral estates (sites 1, 2, 4, 5) where the trees were exposed are grouped on the left, bottom corner of the graph. Assemblages from park complexes and the buffer zones of national parks (site 3 and 6–8) form two distinct groups. Samples from sycamore maples are grouped on the bottom right, while assemblages from lindens and poplars are grouped on the left top corner. The seasons in which the research was conducted did not affect the grouping of the tested samples.

## ﻿Discussion

### ﻿Characteristics of the habitat

Tree barks, thanks to their porosity, can absorb rainwater; therefore the solution on its surface is usually slightly acidic. On the other hand, pH reactions often depend on bark structures, which differ depending on tree species (Zimny 2006). A slightly acidic or close to neutral reaction is typical of the barks of most deciduous tree species, in contrast to conifers, which usually have more acidic barks (Barkman 1958; Steindor et al. 2011; Štifterová and Neustupa 2015). Additionally, the reaction of barks also can decrease with tree age (Grodzińska 1979).

Of the 647 samples collected, only 197 had developed diatom communities, and only single valves were found in the remaining samples. The almost complete or complete absence of diatoms was observed in bark samples covered with visible mats of green algae. Other studies focusing on corticolous algae assemblages, in which only a few species of diatoms have been found (Neustupa and Škaloud 2010; Neustupa and Štifterová 2013; Štifterová and Neustupa 2015), confirm that this microhabitat is not conducive to their development. A similar situation occurred in bark samples covered with lichens. The low number of diatoms in these two microhabitats could be the result of competition for resources with arboreal cyanobacteria and green algae, or it could result from the allelopathic effects of algae and/or lichens on diatoms (Bhattacharyya et al. 2016; Goga et al. 2018).

Although trees growing as close as possible to each other were selected at the sites and materials from analogous microhabitats were collected from them, differences in the frequency of occurrence of diatom assemblages were clearly notable. On bare bark, diatom assemblages were found mainly in materials collected from maples and poplars at locations closest to natural sites (parks and national park buffer zones). However, diatoms were not found or only single specimens were observed in bare bark samples from site 7 (the buffer zone of Magura National Park). This was the only site where samples were collected from aspen poplar (*Populustremula* L.), and it is possible that the bark of this host tree is an unfavorable habitat for diatoms. In urban conditions the trunks of poplars and maples were poorly inhabited by diatoms, and their diversity was concentrated in microhabitats created by bryophytes. Regardless of site, lindens seemed to be unsuitable for diatoms; they were abundant on this host tree only in single samples. The tree species-dependent bark water-holding capacity, which is related to bark features such as stability, texture, thickness, and hardness, directly influenced the intensity of desiccation and were important determinants of the distribution of various organisms overgrowing bark including algae (Valová and Bieleszová 2008; Büdel 2011; Ellis 2012).

Materials from moss microhabitats were more than half of the samples in which large numbers of diatoms were found (123 out of 197 samples). This result is similar to the study by Lindo and Gonzalez (2010) in which the bryophytes overgrowing various substrates (bryosphere), thanks to their structure that permits the accumulation of mineral particles and rainwater, create conditions for the development of different groups of organisms. Only samples of bryophytes from linden and poplar trunks in urban sites, and again from aspen at site 7, contained a few diatoms.

The developed diatom assemblages in the microhabitats analyzed were more often recorded in samples taken from trunk bases than from a height of 150 cm above ground level, and more species were also noted in samples collected from trunk bases (Table 2). This could have been because of the greater availability of moisture from condensation, or it could have been related to the higher share of soil diatoms from the vicinity of the trees. However, the height above ground level did not influence the differences in taxonomic composition between samples from different heights within the tree trunk (Fig. 23).

### ﻿Comparison of novel taxa to similar species

Both of the newly described *Luticola* represent a group of small taxa with elliptic-lanceolate to linear elliptic valves. Many taxa representing this morphological group have been identified previously as *L.mutica*, which is a brackish species (Levkov et al. 2013). Consequently, many modern reports of *L.mutica*, especially those from terrestrial and freshwater habitats, require systematic verification.

*Luticolabryophila* sp. nov. is most similar to the two European species *L.sparsipunctata* and *L.tenuis*. All three taxa share similar sizes and striae densities. The newly described species of the genus *Luticola* commonly shows ghost areolae in the central area, which are absent in both of the other taxa. Additionally, *L.bryophila* can be easily separated from *L.tenuis* based on distal raphe endings, which are short and deflected and not hooked and do not continue onto the valve mantle. *Luticolasparsipunctata* shows two morphotypes with different distal raphe ending morphology. The first of them has a hooked end that continues onto the valve mantle raphe endings, while the second has short and only deflected raphe endings (Levkov et al. 2013, pl. 33). However, *L.sparsipunctata* can also be easily distinguished from *L.bryophila* by the more lanceolate valve shape and the different striae morphology that manifests in the small areolae near the raphe slit and the large areolae near the valve edge (Levkov et al. 2013, p. 222; Fig. 11N), while in *L.bryophila* the areolae in striae are of the same size. Moreover, the internal view of the valve differs in both species. In *L.bryophila* the hymenes form a continuous strip on the striae, while in *L.sparsipunctata* the hymenes are interrupted and cover only the areolae, not the whole striae (Levkov et al. 2013, pl. 33, fig. 7; Fig. 11O).

*Luticolaconfusa* sp. nov. is highly similar in valve and central area shape and striae pattern to *L.imbricata*, *L.pseudoimbricata*, and *L.obscura*. The new species can be distinguished from *L.imbricata* based on its less lanceolate, narrower valves (5–9 µm width in *L.imbricata* vs. 4–6 µm in *L.confusa*) (Levkov et al. 2013, p. 134) and by small depressions on the proximal raphe endings, while they are absent in *L.imbricata* (Levkov et al. 2017, figs 191, 192). Compared to *L.obscura*, which has hooked distal raphe endings terminating on the valve face, *L.confusa* forms hooked distal raphe endings that continue onto the valve mantle (Levkov et al. 2017, fig 169, 170). *L.obscura* also lacks small depressions on the proximal raphe endings, while they are present in *L.confusa*. The structure of raphe endings also distinguishes *L.confusa* from *L.pseudoimbricata*. Both taxa share the same valve dimensions, but *L.pseudoimbricata* has a raphe with slightly deflected proximal endings and distal raphe endings terminate on the valve face (Levkov et al. 2013, pls 16, 1–5).

### ﻿Diversity and composition of the assemblages analyzed

During the study 143 diatom taxa were identified, but most of them were found in single samples and often their share did not exceed 1% of communities. Only 16 species were common in the samples studied (in over 20% of the samples), of which 13 species formed numerous populations (from 10% in assemblages to practically monocultures). The vast majority of the species recorded were taxa commonly identified in various terrestrial environments, mainly in soils (Lund 1946; Ettl and Gärtner 1995; Kerckvoorde et al. 2000; Stanek-Tarkowska et al. 2013; Noga et al. 2014; Barragán et al. 2018; Foets et al. 2021), but also on raised objects like cave walls (Claus 1955, 1964; Krammer and Lange-Bertalot 1991b; Poulíčková and Hašler 2007; Falasco et al. 2014), and on cliffs and walls overgrown by mosses (Round 1957; Casamatta et al. 2002; Lowe et al. 2007; Ress and Lowe 2014).

Assemblages noted on the trunks of all the trees studied growing in city centers and small peripheral estates were dominated by species able to develop in low moisture habitats with high osmotic stress (*Hantzschiaabundans*, *H.amphioxys*, *Humidophilacontenta*, *Pinnulariaborealis*, *P.obscura*) (Lange-Bertalot et al. 2017; Foets et al. 2021). Several co-dominant species were present at these sites, which resulted in higher values of the H’ and J’ indexes.

Similar assemblage structures were also noted on linden and poplar at sites located in suburban park complexes and national park buffer zones. It seems that corticolous assemblages consisting mainly of drought-resistant diatom taxa are typical of these tree species regardless of the degree of tree cover in the area in which they grow. Qin et al. (2016), who studied diatoms inhabiting arboreal mosses (collected from *Cinnamomumcamphora* Ness et Eberm., *Quercusaliena* Blume and *Pterocaryastenoptera* D.CD.) in Wuhan, also found numerous specimens of *P.borealis*, *H.amphioxys*, *O.dendroteres*, and *H.contenta*. These species were also reported in works concerning mainly corticophilic green algae and cyanobacteria, (Neustupa and Škaloud 2010; Neustupa and Štifterová 2013; Štifterová and Neustupa 2015), which indicates that they are common elements of microalgal corticolous biofilms.

Assemblages from sycamore maple (except those from city centers) were distinctly different from those inhabiting linden and poplars because of the strong domination of just one species, which often formed near monocultures (Fig. 23). In parks, assemblages on sycamore were dominated (especially in the case of bryophyte samples) by *Luticolaacidoclinata*, a taxon commonly inhabiting moss clumps even in conditions of high osmotic stress, that are able to survive on highly saline soils (Levkov et al. 2013, 2017). Conversely, on the same host tree species in national park buffer zones with huge tree density, the main dominant species was *Orthoseiradendroteres*, which is typical of more shaded and wet habitats like caves and riparian zones of streams (Poulíčková and Hašler 2007; Škaloud 2009; Czerwik-Marcinkowska and Mrozińska 2011; Czerwik-Marcinkowska et al. 2015; Barragán et al. 2018). The mass development of both taxa resulted in the tremendous homogenization of assemblages reflected in the very low (even 0) values of the H’ and J’ indexes (Fig. 4).

Additionally, many diatom species often common in terrestrial and aerophytic habitats (numerous representatives of the genera *Luticola*, *Mayamaea*, and *Muelleria*; *Microcostatusaerophilus*; *Stauroneisborrichii*; *S.parathermicola*; *S.termicola*; *Sellaphoraharderi*; *S.nana*, *S.subseminulum*) rarely developed in the environments studied. They were either observed in single samples or their share in assemblages did not exceed a few percent (Levkov et al. 2013; Stanek-Tarkowska et al. 2013; Noga et al. 2014; Barragán et al. 2018; Foets et al. 2021). The habitat conditions seemed to be too harsh for the development of these species in larger numbers, or the species could have found suitable conditions on different host trees that were not examined in the current study.

Except for taxa commonly reported from terrestrial habitats, diatoms that usually occur in aquatic environments were noted. The most common were centric taxa such as *Aulacoseira* spp., *Pantocsekiella* sp. and *Cyclostephanosdubius* that are recorded in freshwater planktonic assemblages. They were observed mainly as single, often damaged, frustules, but were observed in 25% of the samples analyzed. Freshwater species associated with epiphytic and epilithic communities (*Achnanthidiumminutissimum*, *Cocconeis* spp., *Gomphonema* spp., *Pseudostaurosirabrevistriata*) were also observed in the materials examined; however, they were noted significantly less frequently than planktonic taxa. Vacht et al. (2014), Martínez-Carreras et al. (2015) and Barragán et al. (2018) also observed typical aquatic diatoms (especially centric ones) in terrestrial environments. Their presence in the environments studied could have resulted from aerosol transmission, which is thought to be the most effective means of dispersing aerosolized microalgal cells, colonies, or whole filaments (Sharma et al. 2007; Harper and McKay 2010; Van Eaton et al. 2013; Sahu and Tangutur 2015; Tesson et al. 2016). Similar natural wind-borne “contamination” in terrestrial habitats was observed by Gottschling et al. (2020), who reported the presence of dinophyte DNA in soil samples. Based on the methods used in the present research, it is not possible to state unequivocally which of the identified species actually live in the habitats examined, only thrive there, or were only empty valves deposited in the bark cracks that were examined.

## ﻿Conclusions

The present research showed that the occurrence of diatom assemblages on tree trunks is influenced by many factors, such as host tree species and the area in which these trees grow, high above soil as well as the presence of suitable microhabitats within trunks. Additionally, diatom assemblage composition was mainly influenced by the tree species.

The current research focused on communities developing on only a few tree species occurring naturally in Europe. Further research involving other tree taxa is necessary for developing a better understanding of corticolous diatom assemblages.

## Supplementary Material

XML Treatment for Luticola bryophila

XML Treatment for Luticola confusa
